# Microplastics in fish and fishmeal: an emerging environmental challenge?

**DOI:** 10.1038/s41598-021-81499-8

**Published:** 2021-01-21

**Authors:** Christina J. Thiele, Malcolm D. Hudson, Andrea E. Russell, Marilin Saluveer, Giovanna Sidaoui-Haddad

**Affiliations:** 1 Faculty of Environmental and Life Sciences, Centre for Environmental Science, University of Southampton, University Road, Southampton, SO17 1BJ UK; 2 Faculty of Engineering and Physical Sciences, School of Chemistry, University of Southampton, University Road, Southampton, SO17 1BJ UK; 3 Present Address: Centre for Environmental Policy, Imperial College London, London, SW7 1NW UK

**Keywords:** Environmental sciences, Ocean sciences

## Abstract

Microplastics are contaminants of emerging concern; they are ingested by marine biota. About a quarter of global marine fish landings is used to produce fishmeal for animal and aquaculture feed. To provide a knowledge foundation for this matrix we reviewed the existing literature for studies of microplastics in fishmeal-relevant species. 55% of studies were deemed unsuitable due to focus on large microplastics (> 1 mm), lack of, or limited contamination control and polymer testing techniques. Overall, fishmeal-relevant species exhibit 0.72 microplastics/individual, with studies generally only assessing digestive organs. We validated a density separation method for effectiveness of microplastic extraction from this medium and assessed two commercial products for microplastics. Recovery rates of a range of dosed microplastics from whitefish fishmeal samples were 71.3 ± 1.2%. Commercial samples contained 123.9 ± 16.5 microplastics per kg of fishmeal—mainly polyethylene—including 52.0 ± 14.0 microfibres—mainly rayon. Concentrations in processed fishmeal seem higher than in captured fish, suggesting potential augmentation during the production process. Based on conservative estimates, over 300 million microplastic particles (mostly < 1 mm) could be released annually to the oceans through marine aquaculture alone. Fishmeal is both a source of microplastics to the environment, and directly exposes organisms for human consumption to these particles.

## Introduction

In recent decades, marine debris composition has seen a shift from natural materials such as seaweeds, shells, pumice and wood floating the oceans to a domination by plastic. Studies suggest that 60–80% of marine debris on shorelines, the seafloor and floating in the oceans consist of plastic^1–5^. All plastic debris can be traced back to human activities, either on land or at sea. Plastic debris ≤ 5000 µm is generally termed microplastic^6^, with the larger fraction (> 1000 µm) being classed as ‘large microplastic’ (ISO/TR 21960:2020). Multiple sources of microplastics exist. Primary microplastics are plastic particles purposefully produced to be of small size either as a raw material or end-product, such as resin pellets for general production of plastic items, and microbeads as abrasives for industrial use and personal care products^7,8^. Secondary microplastics stem from the disintegration of larger plastic items. Such secondary microplastics can be fragments generated through weathering and disintegration processes from plastics already present in the environment, but they can also be microfibres released from laundry or paint flaking off the bottom of marine structures or vessels^7,9–11^. Microplastics have been found in all marine compartments, including biota^8,12^. Trophic transfer has been shown experimentally^13^. The exposure potential of consumers to microplastics is studied by establishing microplastic concentrations in prey and organisms for human consumption. Fish and other marine organisms harvested and cultured for human consumption have been shown to contain microplastics^12,14–17^, but methodological differences can hinder interstudy comparisons. The use of different extraction techniques is often mentioned as such hindrance for comparison. Contamination controls and aspects of polymer identification are rarely scrutinised^18–24^. Effects on organisms such as fish through ingestion of microplastics and associated chemicals have been studied in controlled laboratory experiments, and include hepatic stress, endocrine disruption, behaviour alterations, but numerous studies did not find any effects through microplastic exposure^25–28^. Common criticism of such studies include exposing study organisms to environmentally unrealistic concentrations of microplastics^29^ and the lack of inclusion of control particles for ingestion^30^. Effects of ingestion in wild biota or humans are currently unknown.

Microplastic exposure potential in marine fish, for example, is likely to arise from ingestion of particles in the water column or on the seafloor resembling prey or by ingesting prey that previously ingested microplastics themselves^12^. Aquacultured organisms, including marine fish, are often provided with feed. Fishmeal is often the base for such feeds. Fishmeal is produced from target or bycatch fishing as well as fish by-products^31,32^. About 25% of global commercial marine fisheries landings are destined for the production of fishmeal and fish oil^33^. Fishmeal derivates form part of the human food chain; direct consumption is via pressed fish oil and indirect consumption through feed for poultry, pigs and aquaculture^34,35^. Fishmeal and fish oil are mainly produced with small pelagic species, but also trimmings and wastes from food processing, by-catches and excess of allowable catch quotas^32,33,36–38^ (Table 1). Evidence that such species contain microplastics exists^14–16^.

**Table 1:** Main marine fish species used in fishmeal production, divided into two categories: ‘whole specimens’ and ‘wastes and by-products’.

Main marine fish species for fishmeal production	
**Whole specimens**	
Peruvian, Japanese, European and Southern African anchovy	*Engraulis ringens, E. japonicus, E. encrasicolus & E. capensis*
Blue whiting and Southern blue whiting	*Micromesistius poutassou* and *M. australis*
Sandeels	*Ammodytes* spp.
Gulf and Atlantic menhaden	*Brevoortia patronus* and *B. tyrannus*
European sprat	*Sprattus sprattus*
Kawakawa	*Euthynnus affinis*
Capelin	*Mallotus villosus*
Pacific anchoveta	*Cetengraulis mysticetus*
Atlantic and Mediterranean horse mackerel	*Trachurus trachurus* and *T. mediterraneus*
Pacific sardine, Californian and Southern African pilchard	*Sardinops sagax*
Norway pout	*Trisopterus esmarkii*
**Wastes and by-products**^a^	
Herrings^b^	*Clupea harengus, C. pallasii* and *Strangomera bentincki*
Skipjack tuna	*Katsuwonus pelamis*
Yellowfin tuna^b^	*Thunnus albacares*
Jack and horse mackerel^b^	*Trachurus* spp*.*
Salmonids	*Oncorhynchus* spp*.* and other Salmonidae
Alaska pollock	*Gadus chalcogrammus*
Atlantic cod	*Gadus morhua*
Hakes	*Merluccius* spp*.*
Saithe	*Pollachius virens* and *P. pollachius*
European pilchard^b^	*Sardina pichardus*
Atlantic and chub mackerel^b^	*Scomber scombrus* and *S. japonicus*
Haddock	*Melanogrammus aeglefinus*
Patagonian grenadier	*Macruronus magellanicus*
Pacific thread herring	*Opisthonema* spp*.*
Mote sculpin	*Normanichthys crockeri*
Sardinellas^b^	*Sardinella* spp*.*

Both categories in descending order of overall global catch rates^37,38^. Adapted from^32,33,36–38^.

^a^By-products (e.g. offal) and other wastes from food processing industry, fisheries discards or excess from total allowable catch from prime food fish.

^b^At least some of whole fish catch to fishmeal reduction.

Evidence about microplastics in fishmeal is only just starting to emerge. The research aims of this present study are threefold: (1) to review the existing literature of microplastics in fishmeal-relevant fish species to establish the potential contribution of the raw material to fishmeal, including critically examining the methods used; (2) to establish a suitable method to extract small microplastics from fishmeal. To our knowledge, two studies have investigated this matrix to date: both studies focus on microplastics ≥ 149 µm^39,40^. By extending the size range, a more accurate picture of microplastic contamination in fishmeal should be obtained. (3) To test such method on commercially available samples to establish if microplastics in fishmeal should be a concern. The results will improve our understanding of the potential for microplastics to enter the (human) food chain via fishmeal and inform future assessments of associated risk to health and food security.

## Results

### Review of relevant studies: microplastics in fish that are used in fishmeal production

#### General methods

Twenty-nine studies investigating microplastics in fishmeal-relevant fish were included in this review; 34 were excluded after applying criteria outlined in the methods section. Detailed information on individual studies can be found in Supplementary Table S1. Studies were published between 2013 and 2020. None of the studies assessed whole body specimens. Twenty-six assessed the gastrointestinal tract or parts thereof, one investigated the gastrointestinal tract and gills and two the gastrointestinal tract, gills and other organs (one of the latter also included 5 g subsamples of muscle). Most studies used extraction techniques to isolate microplastics except for nine studies. KOH was most commonly used (12 studies, three of which further performed density separation). HNO_3_ or NaClO/HNO_3_ was used by two studies, while Proteinase-K and H_2_O_2_ and a NaCl density separation without additional digestion were used once each. In most cases, reagents are not filtered prior to use (see Supplementary Table S1 for detailed information).

#### Polymer identification techniques and assessment rates

The most used polymer identification technique was Fourier-transform infrared (FTIR) spectroscopy (25 studies), three used Raman spectroscopy and one study combined both. Of the excluded studies, 18 studies did not apply any chemical identification technique, seven did not state either how many of the potential microplastics were assessed or they assessed less than 10% by FTIR. A range of spectral libraries were used for positive identification of plastics. Of the included 29 studies, 16 did not report a minimum match score for positive identification against library searches. One study used a minimum score of 60% and nine used 70%, of which three visually assessed the spectral comparison results when the score was ≥ 60%. The remaining studies set the score to 80% (one study) and 85% (two studies). Twenty-six studies reported detailed information of how many potential microplastics isolated from fish were assessed with spectroscopy. These studies assessed on average 62.6% (± 37.3%) of potential microplastics—ranging 10–100%. The lowest quantity assessed was two, which was 100% of particles found; the highest was 2649 particles, equalling 77% of potential microplastics found. Interestingly, 66.6% of the lowest spectroscopy assessment rates (i.e. < 50% of particles assessed) coincided with a low number of absolute particles assessed (assessment rates of 10–36% equalling assessment of 3–38 potential microplastics). Eleven studies provided an insight into how many particles proved to be plastic (one of these studies reported values individually per both species analysed). Outcomes were variable: mean 66.6% (± 24.4%), ranging 31–94% of potential microplastics positively being identified as plastics.

#### Target size

Seventeen studies assessed microplastics < 100 µm, two had a limit of detection (LOD) or filter size between 100 and 200 µm and one assessed particles ≥ 500 µm. Nine studies did not state the filter size or LOD size of their method. Of the studies that did not report LOD/filter size, four also did not report the sizes of the microplastics they presented in their results. Of the studies with LOD/filter size < 100 µm, seven studies reported finding particles < 50 µm.

#### Microplastic categories

Four studies did not report microplastics categories. Concentrating on the fibre category (due to its relevance to airborne contamination^16,41–44^), only three studies reported no microfibres, three reported occurrences of 6–20%, three 33–42%, ten studies 55–70% and the remaining thirteen ≥ 80, with two reporting 100% (since some studies reported fibre proportions per individual species, the overall sum here is 36).

#### Contamination control

The use of airborne controls in the absence of cleanrooms or laminar flow cabinets was reported by eight studies, of which two did not report any fibres. The remaining studies reported that 33, 56, 62, 80 and 96% of all microplastics found were fibres. Six studies used a laminar flow cabinet and provided enough information on categories; of those, two did not find/report any fibres and the remaining ones had fibre percentages of 16, 42, 60 and 80% each. Only one study was performed in a clean room, and incidentally only found fibres. Using the most stringent contamination control by performing airborne controls while also using a laminar flow cabinet, the only study doing this reported 16% fibres.

#### Microplastic concentrations in fishmeal-relevant marine fish

**Figure 1:**
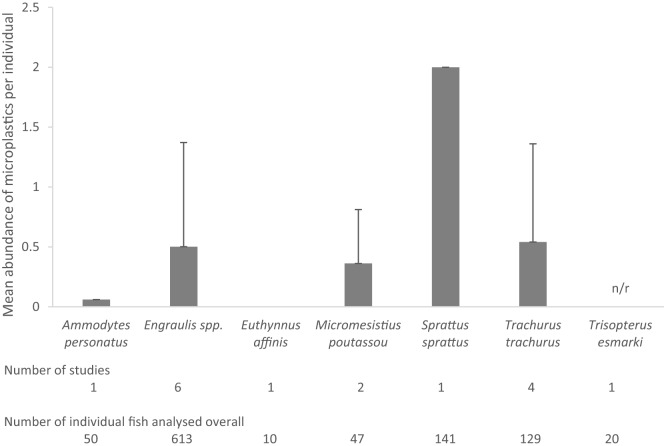
Mean abundance of microplastics per individual in studies assessing microplastics in fishmeal-relevant fish species. Only concentrations of ‘whole fish’ species used in fishmeal production are shown; 12 reviewed studies examined those species—four of those analysed two of such species each. *n/r* mean abundance was not reported. Error bars are 1× standard deviation of mean abundance per species of each study.

Detailed information per species can be found in Supplementary Tables S2 and S3. Fifty-six species were assessed, some—such as *Engraulis encrasicolus*, *E. japonicus*, and *Trachurus trachurus*—were studied more than once. Of the ‘whole specimens’ category for fishmeal production (Table 1), *E. encrasicolus* and *T. trachurus* were the most studied (four times each). Of the ‘wastes and by-product’ category, *Sardina pilchardus* was the most studied (5), followed by *Decapterus* spp., *Gadus morhua, Mugil cephalus, Scomber* spp. (studied each 4 times). Overall, 1010 specimens of ‘whole specimens’ for fishmeal species were analysed for microplastics, but for the 20 *Trisopterus esmarkii* abundance was not reported. Mean abundance of microplastics in fish destined whole for fishmeal production was 0.69 ± 0.81 items/specimen, ranging 0–2 items when broken down per species (Fig. 1). In the ‘wastes and by-product’ category, 1713 specimens were analysed, but microplastic abundance was not reported for 92 specimens. For the remaining, mean microplastic abundance was 0.73 ± 1.61 items/specimen. Pooling all studies, mean microplastic concentrations based on 2611 specimens was 0.72 items/individual. Highest mean microplastic abundances per study were reported for *E. japonicus* with 2.3 items/individual (‘whole specimen’ category) and *Muraenesox cirereus* with 7 items/individual (‘wastes and by-products’ category). As elaborated in results in “General methods” of the “Review of relevant studies: microplastics in fish that are used in fishmeal production” section, concentrations per individual usually refer to concentrations in digestive organs. While individual studies reported absence of microplastics in their studied species, when more than one study was performed per species, usually there was only one study reporting zero concentrations with the exception of *Clupea harengus* (2 out of 3 studies did not find microplastics) and *Gadus morhua* (3 out of 4 reported nil microplastics). Based on the review of species destined whole to fishmeal production, approximately 36 microplastics per kilogram of fishmeal can be expected from the raw material.

### Method development and assessment for extraction of microplastics from fishmeal: NaCl density separation and recovery rates

#### Technique 1

Filtration of fishmeal digestates (exposed to 10% KOH) was not achieved using 25-µm filters. Supernatants of density separation (using NaCl) were successfully filtered over 25 µm. Overall microplastics recovery rate of dosed samples was 60.3 ± 12.1%, but with a variation of an order of magnitude lower within each fishmeal type. The overall recovery rate from dosing trials with whitefish fishmeal was 71.3 ± 1.2%, ranging 46.7% for polypropylene fragments to 100.0% for polystyrene fragments (see Supplementary Table S5 for recovery rates per polymer type). Microplastic recovery from sardine/anchovy fishmeal was 49.3 ± 1.2%, ranging 30.0% for PET and rayon fibres to 76.7% for polystyrene fragments. By polymer density, 49.2 ± 25.0% of particles of a density > 1.2 g/ml were recovered. Overall, 48.7% of dosed particles were recovered during overflow #1, 7.3% with overflow #2 and 4.3% with overflow #3.

#### Technique 2

Recovery rates of microplastics using the Sediment-Microplastic Isolation unit were 31.9 ± 8.3%, with recovery rates from whitefish being 38.3 ± 2.0% and from sardine/anchovy fishmeal 25.6 ± 6.9%.

### Microplastics in commercial fishmeal samples

**Figure 2:**
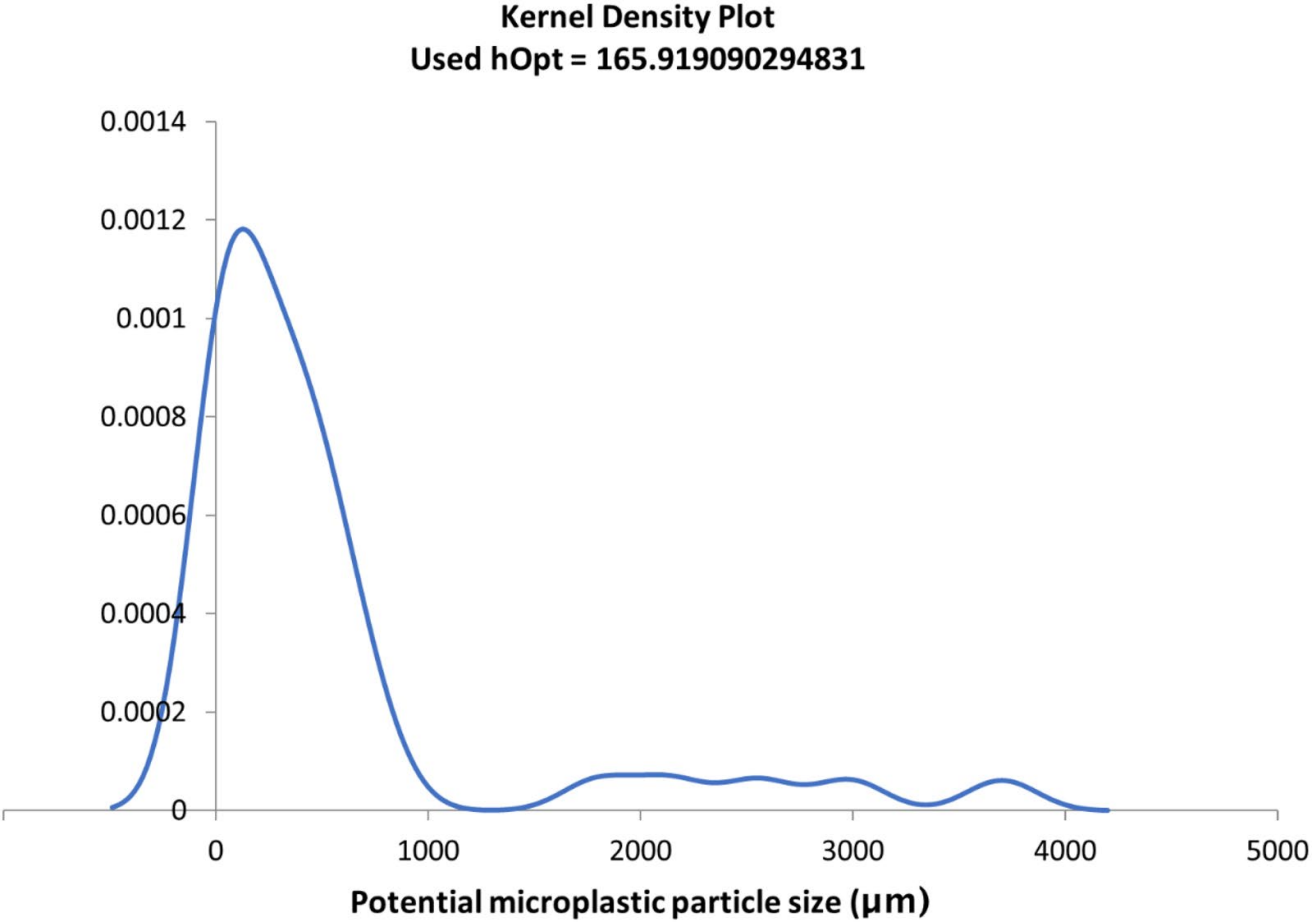
Kernel density estimation of potential microplastic size distribution based on 40 out of 58 particles extracted with NaCl density separation from whitefish fishmeal (n = 6). 87.2% were microparticles ≤ 1000 µm and 12.8% microparticles 1000–5000 µm. Note: the single > 5000 µm particle (6436 µm) is excluded from microplastic calculations.

Procedural blanks were free from potential microplastics. Airborne controls (dampened filter papers, n = 4) contained in total seven fibres, which had accumulated during the processing of 24 fishmeal/procedural blank filters. Since numbers are low, fibres are not discounted from samples, but all 14 fibres found in the fishmeal samples are presented separately. 57 potential microplastics were visually identified (including 14 fibres), plus one fragment > 5000 µm (6436 µm). Fragments and microsheet extracted with this method ranged 56–3701 µm, fibre diameters were 12–57 µm and fibre length approximately 76 to 3200 µm. Size distribution was estimated (Fig. 2). Mean fragment size, excluding the 6436-µm particle, was 778 ± 944 µm (median 408 µm), mean fibre diameter was 24 ± 15.3 µm (median 16 µm) and mean length 1299 ± 935 µm (median 1200 µm).

Approximately 90% of potential microplastics (52; including approximately 23% of fibres) were assessed with Raman spectroscopy. Of these, no spectrum could be achieved for 15 particles (none of which were fibres). Eleven particles were confirmed to be biomaterials, such as tricalcium phosphate. Three fibres were of natural origin. Nineteen potential microplastics were confirmed to be plastic (plus eight fibres). The 6436-µm particle was also plastic. Based on this and corrected for recovery rates of 71.3% and spectroscopy assessment rate of 89.1%, the mean concentration of non-fibrous microplastics per kg of fishmeal is 71.9 ± 2.5 items. Mean microfibre concentrations were 52.0 ± 14.0 fibres/kg fishmeal. Concentrations of ‘inconclusive’ non-fibrous particles (mainly identified as dyes) were 6.4 ± 9.1 items and for fibres 18.1 ± 25.6 items.

**Figure 3:**
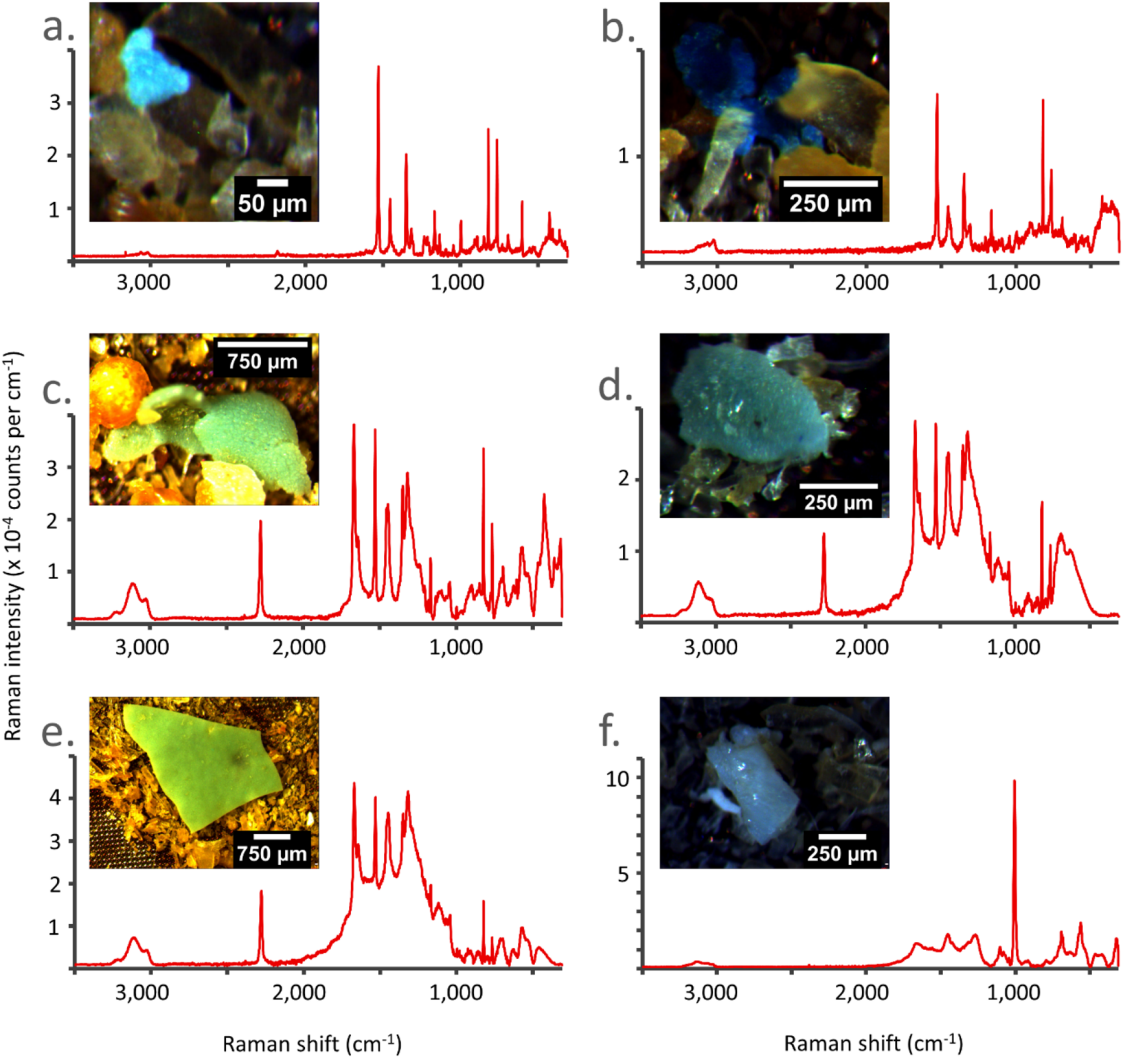
Identification of six particles found in fishmeal samples. (**a**,**b**) Microfilms identified as acrylonitrile butadiene styrene—match score of 88.0 and 93.3% respectively, (**c**,**d**) polyethylene microfilms—match score of 86.3 and 86.1% respectively, (**e**) acrylonitrile/butadiene/styrene resin or styrene/acrylonitrile copolymer fragment with blue dye—match score 90.2%, (**f**) fragment of biomaterial (top suggestion: calcium phosphate—match score 93.6%).

Microplastic categories in fishmeal were 21.1% fragments, 36.8% microsheet/film and 42.1% microfibres. Of non-fibrous microplastics, 54.5% were made of polyethylene (Fig. 3a,b), 18.2% acrylonitrile butadiene styrene (Fig. 3c,d) and 9.1% each nylon, PET/polyester and the other likely to be acrylonitrile/butadiene/styrene resin or styrene/acrylonitrile copolymer (Fig. 3e). Of microfibres, 62.5% were rayon, 12.5% each nylon, PET/polyester and polypropylene. Non-fibrous microplastics in fishmeal were predominantly blue, followed by white and red. Microfibres were blue and black. Potential microplastics that were not confirmed to be so, included a white fragment (Fig. 3f) but also numerous orange spheroids (see next to microsheet in Fig. 3c). No spectra could be obtained of the latter. The only plastic particle larger than a microplastic was a blue LDPE film. Mean match score was 87%. The lowest score of 61% was obtained through peak matching, all remaining scores were > 70% and obtained through component analysis.

## Discussion

Here we present a suitable extraction technique for microplastics from fishmeal. Our review has shown that potassium hydroxide digestions are regularly used for microplastic extractions from gastrointestinal fish tissues. KOH has also been used for fishmeal digestions^39,40^, but recovering microplastics < 150 µm does not seem possible for fishmeal with this method. The investigation of small microplastics is important, but previously often neglected. For one, smaller particles may be more prevalent. For example, 80% of microplastics in *E. japonicus* were 150–1000 µm and all microplastics found in *S. pilchardus* were 39–857 µm^15,17^. Across numerous species, Bour et al*.*^42^ found 12% of microplastics 41–100 µm and 47% 100–200 µm. Secondly, smaller particles may be more likely to translocate to other tissues^45^—this has been shown in bivalve models^46^ but also observed in wild fish liver^47^. We successfully trialled NaCl flotation to extract microplastics from fishmeal. A simple overflowing technique provided the highest recovery rates of spiked microplastics; 35% of potential microplastics in our samples were < 150 µm highlighting the importance of recovering smaller microplastics and the suitability of our method. Flotation recovery rates were lower than they are for digestions^21,23^, but still averaged 60% and up to 100% for certain polymers depending on the type of fishmeal. Even polymers of greater density than the NaCl solution (e.g. PET/polyester, rayon) were extracted with our technique. In addition, low standard deviations within the same type of fishmeal suggest high repeatability of recovery rates.

We identified numerous limitations in studies investigating microplastics in fish. Over half of the initially qualifying studies were excluded for three reasons: focus on large microplastics (i.e. > 1 mm), lack of polymer identification techniques or limited identification assessment rates. Knowledge about ‘true’ microplastics (≤ 1 mm in line with ISO/TR 21960:2020) is imperative considering their prevalence and potential hazardousness as elaborated above. Lack, or limited use of stringent confirmatory techniques is a reoccurring issue in microplastics research. Our review has highlighted that spectroscopy assessment rates in many previous studies might be an issue. Numerous studies were excluded from the review for not testing polymer composition at all, despite reporting ‘microplastics’. Often, when small numbers of potential microplastics were found, only a small fraction of these (typically ≤ 38 particles) were tested. Polymer identification is important, especially to reduce overestimates of potential microplastic counts. Based on the review and our own study, as little as 1/3 of potential particles may prove to be plastic, averaging a confirmation rate of 67%. For this reason, it is imperative to conduct spectroscopy or other polymer identification methods. In the absence of automated or semi-automated systems, a subset of at least 50 particles and all particles 20–100 µm should be analysed to confirm their polymeric identity—as previously recommended by Galgani et al*.*^19^.

A further issue relates to confirming spectral library search results. Match scores, a computation of the quality of similarity between the spectrum of a potential microplastic against spectra from chosen libraries based on a Hit Quality Index and scaled up to 100^48,49^, are generally used to confirm polymeric identity. Of the studies that provided such information, all set the minimum match score to 60% or higher. Generally in spectroscopy, > 80% would be considered a high quality match and 50–80% a medium quality match^48^. Medium quality matches can be expected from environmental microplastics often exhibiting poor/degraded spectral quality (personal observation). No matter the score, Smith^48^ suggests to visually compare all results. This was only done by three studies, and only for the match range 60–70%. To improve interstudy comparability we recommend reporting of particle assessment rates, which spectral libraries were used, and the minimum score applied to obtain a positive identification. Such information is as vital as reporting contamination mitigation techniques and will aid comparison between studies.

Our results show that microplastic is present in our samples, and that concentrations in fishmeal are low and solely seem to consist of secondary microplastics. Based on the review of fishmeal-relevant fish species, mean microplastic concentrations are 0.7 per individual and could, therefore, be as little as 36 microplastics/kg (mean minimum size assessed 146 µm) in fishmeal made with fish destined whole to fishmeal production. When waste products such as gastrointestinal tracts are included and minimum particle sizes assessed are < 146 µm, microplastic concentrations may well be higher. It seems that concentrations in commercial fishmeal (even when not including any microfibres) are higher than in the raw material. This observation can be extended to other recent fishmeal studies. Karbalaei et al.^40^ investigated three types of fishmeal. The fishmeal produced with whole specimens of Indian mackerel (*Rastrelliger kanagurta*) contained 200–300 microplastics per kg^40^. Hanachi et al*.*^39^ analysed salmon, sardine and kilka fishmeal from Iran. They reported approximately 4000–6000 microplastics per kg^39^. Microplastic concentrations of these species are listed in Supplementary Table S3, and as with our samples, the raw material seems to contain lower concentrations of microplastics. This overall pattern of increased microplastic concentrations from wild caught fish to fishmeal products warrants further investigation: production processes could lead to fragmentation of existing microplastics in the raw material, and/or product manufacturing, handling and storage may be introducing microplastic contamination to the end-product. Microfibres could potentially enter the production process at any point. This is supported by widespread microfibre contamination in the atmosphere^41,43,44,50^. Fragments or films may enter with the raw material, or in case of the latter, also during storage of the final product. Most of the microplastics we found were polyethylene—a material used to make storage bags for fishmeal^31,39^.

Fishmeal samples analysed here contained about half the microplastic concentrations found by Karbalaei et al*.*^40^ and even less than the quantities reported by Hanachi et al*.*^39^. Methodological differences are unlikely to result in such a discrepancy. Those studies digested samples with KOH^39,40^. Recovery rates from biological tissues using KOH have been reported as > 93% from whole fish and 86.2% (± 7.9%) from bivalve tissues^21,23^, therefore higher than recovery rates achieved here. However, while we applied the recovery rates as a correction factor to our results, others did not. Further, using a pore size of 149 µm^39,40^, smaller particles are likely to have been lost. We recovered three fragments and eleven fibres < 150 µm. It might therefore be that either the fish in the other studies or parts thereof contained higher microplastic concentrations at the time of harvest, that microplastic contamination was introduced during the production processes or more aggressive physical forces were used in pressing and grinding leading to fragmentation of microplastics that were present. It is therefore paramount to conduct additional research into microplastics in fishmeal to help understand those discrepancies between studies, in addition to differences between raw materials and end products.

**Figure 4:**
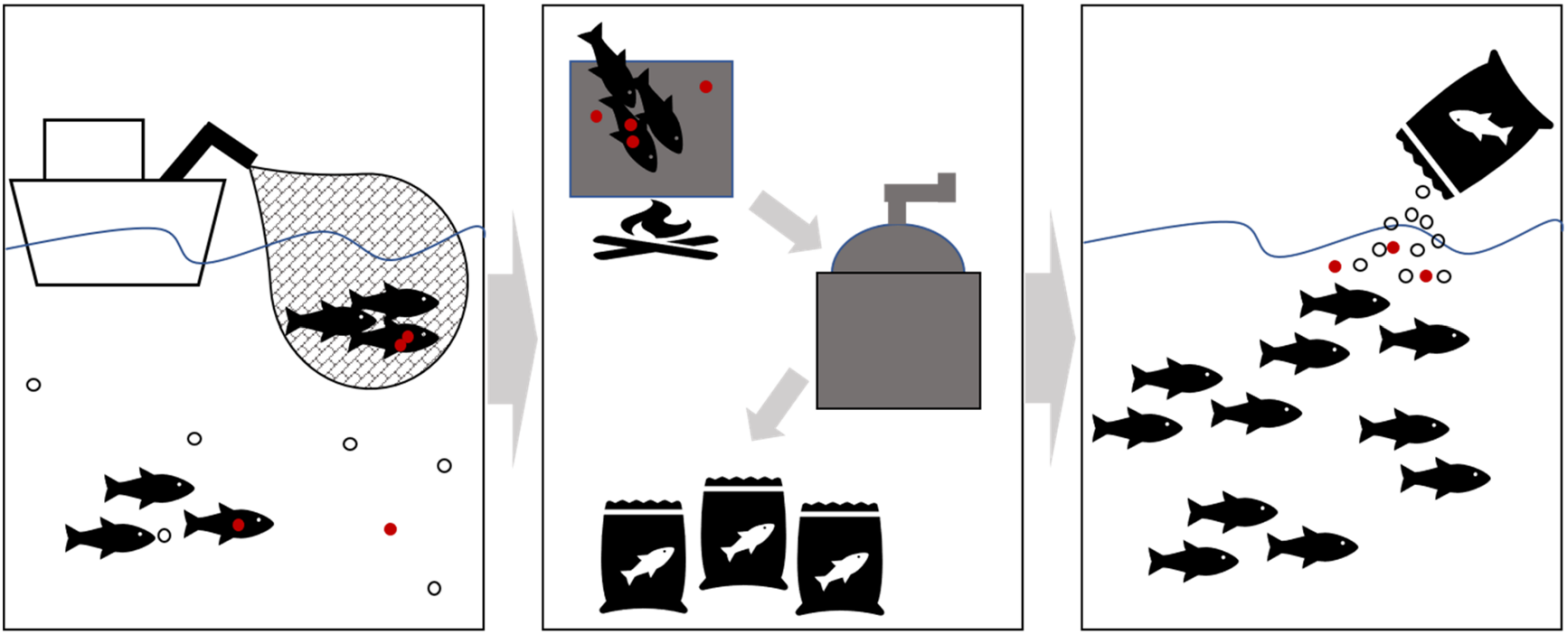
Fishmeal as a pathway for microplastics to the marine environment from capture fisheries via fishmeal production to mariculture feed. Food particles (open circle), microplastics (red filled circle). Microplastic fragmentation possible during fishmeal production steps (heating and grinding) or through contamination, leading to increased microplastic concentrations fed back into the environment.

Fishmeal is a pathway for microplastics into the environment (partly returning microplastics that were previously taken out, but also potentially adding new ones) since a proportion of fishmeal is thrown into the sea as aquaculture feed (Fig. 4). Based on the application of 2.5 million tonnes of fishmeal per year for marine aquaculture^33^, currently about 180–310 million pieces of microplastics might be put into the oceans per year and may equate to adding 10–1670 kg of microplastics annually (see Supplementary Section “Quantification of theoretical microplastic concentrations in fishmeal online for calculations”). These global extrapolations may be a relatively small, compared to 65 million microplastics being released daily from individual wastewater treatment plants^11^ for example. However, based on our results almost 90% of those microplastics could be < 1 mm in size. Furthermore, fishmeal is also used in freshwater aquaculture and fish farms, including lakes^32^. Also, the application is intensive at aquaculture sites as many of which are in low energy coastal waters or lochs where dispersal may be limited.

In addition to simply increasing microplastic concentrations in the environment, the microplastic exposure potential is increased. Fishmeal is an integral part of the food chain by being fed to poultry, pigs and aquaculture amongst others^31^. Hence, these organisms are directly exposed to the microplastics mixed with their feed. A laboratory study has already confirmed ingestion of microplastics present in fishmeal in *Cyprinus carpio*^39^. However, microplastic residence time in fish is likely to be short. Bråte et al*.*^51^ found that fish with empty stomachs did not have large microplastics in their system. Ingested plastic particles may often pass through without harming the organism visibly^52^. Also, since particle size of encountered microplastics is in the range of prey items and fishmeal particles, such particles may be processed by the digestive systems in a comparable fashion. Similarly, chickens (*Gallus gallus domesticus*) are known to ingest microplastics^53^. Huerta Lwanga et al.^53^ found microplastics ranging 100–1000 µm in chicken faeces, and larger particles in gizzards and chicken crops. Only finding smaller particles in faeces, but larger in crop and gizzards, suggests that particles > 1000 µm may not easily pass through the digestive system of chickens. We only found low numbers of potential microplastics above this size. The largest microplastic found by others in fishmeal was 810 µm^39^. Based on the evidence to date, microplastics from fishmeal are therefore likely to pass through chickens’ intestines. Chicken manure is widely used as a soil improver^54^, posing the question where those microplastics might go next. There is currently no evidence to suggest that microplastics in fishmeal could threaten food security in any way. The presence of microplastics in fishmeal does have limited implications for the human food chain; namely the theoretical potential of small microplastics and nanoplastics translocating into edible parts of organisms consumed by humans and fed with fishmeal or exposure to chemicals innate or adhering to microplastics. The latter implication is currently mainly based on extrapolated knowledge from macroplastics to microplastics^55^. For both cases, more research is needed to establish if microplastics or chemical exposure related to those particles could harm animals feeding on fishmeal, especially because we have limited knowledge about particles > 55 µm in fishmeal.

Some potential limitations should be highlighted. Estimated microplastic concentrations that can theoretically be expected in fishmeal based on our review of fishmeal-relevant fish species might be an underestimate. This is because fish weight was often not provided and because most studies investigate microplastics in the digestive system rather than whole organisms. The gastrointestinal tract, which is removed prior to human consumption—except for small pelagic fish eaten whole or without the head, is increasingly processed together with trimmings and other waste products such as organs for fishmeal^31,34,35^. Evidence of microplastic presence in tissues outside the digestive system is slowly emerging. Microplastics have been found in the gills of a number of fish species^56,57^. They have further been found in the livers of European anchovies (*Engraulis encrasicolus*), but not in the livers or muscle of a number of other important commercial species^47,56–59^. Sample quantities may not have be adequate, however; Su et al*.*^58^ assessed subsamples of livers and muscle of approximately 0.7 g and 3.1 g respectively and Huang et al*.*^57^ assessed tissue subsamples of 5 g. Microplastics in fish muscle have been reported^60^. However, said study did not perform any polymer identification techniques. The global estimate of microplastic concentrations entering the oceans through fishmeal is likely to be an underestimate as well. These calculations were based on our findings in whitefish fishmeal, which even after correcting for recovery rates, were lower than concentrations reported in other studies.

We acknowledge that the use of visual particle assessment and manual Raman spectroscopy are not ideal, as this potentially leads to a proportion of particles being overlooked^61^. However, one aim of our study was to establish if microplastics are present in fishmeal. The visual assessment/manual Raman spectroscopy approach was adequate for this, and we exceeded practices in many recently published studies by testing the large majority (89%) of the microparticles we could isolate. Furthermore, the visual assessment/manual spectroscopy combination was also applied in all studies we reviewed. About 62% of the suspected microplastics from the visual assessment were not plastic. This is in line with previously reported numbers^18^.

Our work has highlighted that despite stringent controls, atmospheric contamination may not be avoidable. Numerous studies reviewed here conducted their work in laminar flow cabinets, but only one of those also implemented airborne controls—leading to one of the lowest microfibre proportions of 16%^62^. For this reason, using airborne contamination monitoring such as dampened filter papers should be considered even in clean environments. Lastly, while we were successful at extracting microplastics from whitefish fishmeal samples with our extraction technique, this method might not be suitable for all types of fishmeal, and further method development is recommended. Despite these limitations our research aims were fulfilled.

## Conclusion

Microplastic extraction from whitefish fishmeal using a simple NaCl density separation is suitable for fragments/sheets > 55 µm and microfibres with a diameter smaller than this. Fishmeal is a small, yet important source of microplastics to the environment—especially to low energy environments already affected by aquaculture practices. Further research is needed to enable extraction of particles to 1 µm and to improve applicability to further fishmeal types. More work is needed to understand the relationship of microplastics in capture fish and fishmeal, and the implications of their presence for direct and indirect consumers of fishmeal given the importance of fishmeal for food security. Reviewing research articles of fishmeal-relevant fish species has highlighted issues, such as a lack of transparency regarding aspects of microplastic identification, that are often ignored but obstruct interstudy comparability. To improve comparability it is imperative to provide size-related information of extracted particles; we also recommend that studies report the proportions of potential microplastics that were assessed using polymer identification techniques (in the absence of automated systems) and the minimum scores for positive identification against spectral library searches that were applied. Lastly, based on our and the reviewed work we suggest the use of airborne contamination monitoring, even in clean environments.

## Methods

### Review of relevant studies: microplastics in fish that are used in fishmeal production

A Web of Science search (terms “marine AND micro$plastic* AND fish”) was conducted on 18/03/2020 yielding 560 results. Only research articles investigating microplastic concentrations in adult fish that are of interest for fishmeal (see Supplementary Table S6) were preselected. Of the 65 remaining studies, inclusion in the review was subject to (1) use of polymer identification technique, such as Raman spectroscopy, and assessment of at least 10% of potential microplastics, (2) focus on ‘true’ microplastics, i.e. target size range including particles < 1000 µm and (3) reporting of at least some contamination control. The rationale for the size exclusion was that numerous studies concentrate on larger microplastics despite extensive evidence of large proportions of microplastics found in fish  being < 1000 µm^15,17,42,57,63,64^: Twenty-nine studies were included in this review (Supplementary Table S1). Some studies investigated numerous species, but only records of fishmeal-relevant species were processed for the microplastic concentrations assessment (Supplementary Tables S2, S3).

Manipulations of results from review studies were performed as follows: Mean values were reported unless otherwise stated. When concern about potential contamination with fibres existed based on the reported methods in the respective studies (if airborne controls were not performed or when performed but no further contamination mitigation was reported), microplastic concentrations were adjusted by subtracting fibrous microplastics from results when enough detail was provided by those studies. Mean overall microplastic abundances were calculated by obtaining total microplastic concentrations (mean reported or adjusted concentration × number of specimens of each study) and dividing those results by the total number of specimens of all studies. Standard deviations were calculated based on the mean abundance per species of each study. Theoretical concentrations of microplastics that could be anticipated in fishmeal samples were calculated based on information available of fishmeal-relevant species that are destined whole for production, detail of these calculations can be found in Supplementary Section “Quantification of theoretical microplastic concentrations in fishmeal”.

### Method development and assessment for extraction of microplastics from fishmeal

#### Materials

Method development was performed with two types of fishmeal (whitefish and sardine/anchovies). Whitefish fishmeal was obtained from two suppliers for quantification of microplastic concentrations. Samples of 40 g were used.

#### Method development background

Numerous studies digested fish tissue with 10% KOH^15,16,21^. Visual or molecular damage to microplastics (hindering spectroscopy to establish polymer compositions) has been found to be minimal from exposure to KOH^21,23^. KOH is often recommended as the most suitable digestion method of biotic materials^16,21,23,65–67^. However, preliminarily trials revealed that 10% KOH (1.82 M; 3× v/v, digested at 60 °C for 48 h) was not able to digest fishmeal enough to be vacuum-filtered over 25-µm filters. This filter size was chosen to allow catching at least a proportion of small particles (< 150 µm) that could potentially harm consumers due to their size^45^. Compared to the content of fish guts, fishmeal may contain higher concentrations of lipids and have more very fine fragments of bone from the grinding/milling process making the samples difficult to filter. The application of an acid reagent may digest those bony fragments; however, as previous comparisons between extraction reagents have shown, microplastic particles are likely to be compromised by this approach^68^. Since fishmeal is a matrix of small solid particles, density separation techniques were explored. Microplastics are often retrieved from sediment samples by means of flotation using brine solutions^18,69,70^. Saline solutions are more dense than water and therefore allow for a greater fraction of particles (i.e. with a lower density than the solution) to float to the surface and be removed, leaving heavier particles such as bones, shells and sediment behind^18^. Flotation with NaCl was successfully trialled as follows.

#### Sodium chloride density separations

*Technique 1* Samples of 40 g fishmeal were weighed out into 400-ml glass jars. Approximately 3× the volume of the sample of saturated NaCl solution was added, almost filling the jar. Care was taken to not fill the jar to the top but keep a gap of approximately 10 mm between the liquid and the lid. This was done to ensure the same amount of liquid to be collected during each overflow. Lidded sample jars were shaken vigorously for 15 s and subsequently left to stand for a minimum of 30 min. Jars were placed into a large beaker and saturated NaCl solution (1.20 g cm^3^, filtered through 1.2 µm GF/C filters) carefully poured into the jar until the solution overflowed (see Supplementary Fig. S1 for setup). The outside of the jars were rinsed with copious amounts of ultrapure water. Supernatants were vacuum-filtered, and filters dried overnight at 40 °C in closed petri dishes. Each sample was subjected to three extractions. *Technique 2* Further, the Sediment-Microplastic Isolation unit developed by Coppock et al.^71^ was assessed. Their method was adapted as follows: 40 g fishmeal were weight out into the unit and approximately 540 ml of saturated NaCl solution was added. The sample was stirred with a glass rod. The unit was placed on a magnetic stirrer for 5 min and left to stand for a further 5 min. The sample was stirred again with a glass rod and the procedure repeated. This was to ensure wetting and total mixing of the fishmeal with the solution. The glass rod was rinsed each time with approximately 40 ml of NaCl solution which was added to the sample. The unit was then left to stand for a further 40 min before the tap was closed. Different standing time compared to technique 1 was needed because fishmeal settled at a slower rate in the isolation unit, potentially because the fishmeal was stirred rather than shaken in the NaCl solution. The supernatant was poured into a glass jar, and the top part of the unit was rinsed with 200 ml of NaCl solution, which was also transferred to the jar. The extraction procedure was performed three times per sample.

### Recovery rates

Microplastics were created from five post-consumer items (mean size 298 ± 201 µm). Fragments of polystyrene (PS; lid of disposable coffee cup, white), polypropylene (PP; single-use ready meal container, transparent) and polyethylene terephthalate (PET; bottle of cooking oil, green) were produced with an electrical coffee bean grinder. Particles were dry-sieved through stacked 63 and 600-µm stainless steel sieves, retaining microplastics on the 63-µm sieve. Fibres of nylon (PA; tutu fabric, yellow) and rayon (garment fabric, black) were cut from sheets of fabric. A subsample 50% of the created particles was measured using a Nikon SMZ1000 microscope equipped with a graticule. Ten particles of each material were added to triplicates of two types of fishmeal (sardine/anchovies and whitefish). The above-mentioned density separation techniques were used. For technique 1, four procedural blanks were run alongside the extraction process and vacuum-filtration was performed using cellulose filter papers, grade 4 (25 µm). For technique 2, two procedural blanks were run alongside, and supernatants were filtered over 55-µm aperture metal mesh. Recovered microplastics were manually counted using a Nikon SMZ1000 microscope. Number of recovered microplastics was expressed as a percentage of dosed particles.

### Microplastics in commercial fishmeal samples

#### Extraction and enumeration

Due to filtration issues when not taking absolute care during the overflow step, supernatants were filtered through 55-µm aperture metal mesh disks (diameter 47 mm). Whitefish fishmeal from two suppliers in triplicates were subjected to the above-mentioned extraction method. Without removing the lids, potential microplastics (in line with Hidalgo-Ruz et al*.*^18^ and MERI^72^) were manually counted on the filters using a Nikon SMZ1000 microscope (magnification ×10–×80) with attached CMEX500 digital camera. Particles were photographed and dimensions measured in ImageJ^73^. Particles were then transferred onto 1.2-μm GF/C filters for subsequent Raman analysis. Findings are expressed in means ± standard deviation unless otherwise stated. Background information about calculating global annual microplastics input into the oceans through fishmeal can be found in Supplementary Section “Quantification of fishmeal as microplastics pathway to the sea”. Kernel density estimation was performed with the Excel Add-In of the Royal Society of Chemistry^74^ and figures drawn in Microsoft Excel.

### Particle composition verification

Potential microplastics were analysed using Raman spectroscopy (Renishaw inVia, excitation wavelength 785 nm, reproducibility < 1 cm^−1^, absolute power ≥ 300 mW, with Leica DM 25,000 M microscope, ×50 magnification lens, WiRE 4.1 software). Particles were manually selected. Analysis was performed over the entire spectrum (Raman shift 0–3200 cm^−1^) with 1–100% laser power, 10 s exposure time and three accumulations. Particle spectra were compared against our own Raman polymer library, SLoPP(e)^24^ and standard libraries in BioRad KnowItAll. Minimum acceptable scores were 70% for individual and multi-components results and 50% for peak results, all scores were also visually assessed. Particles were classed as ‘inconclusive’ when only dyes could be identified.

#### Contamination control

Researchers wore white 100% cotton clothing (headscarf, overall and laboratory coat). Clothing was lint-rolled prior to laboratory work each day to minimise the risk of sample contamination with fibres. Work surfaces were wiped down 3× with 70% ethanol. Work was performed in a clean air cabinet (Bassaire 03VB, BS EN ISO14644, class 5, with additional cover), except for microscopy work. Glass and metal ware were used whenever possible. GF/C filter paper and mesh were furnaced at 500 °C for two hours to remove potential plastic contaminants and stored in airtight containers until use. NaCl solutions were filtered over 1.2-μm GF/C filters and stored in glass bottles with glass lids until needed. Dampened filter papers (1.2-μm GF/C) were placed on work surfaces to assess potential airborne contamination in the clean air cabinet. Two procedural blanks were run alongside the samples and handled the same way as the samples. Blanks were sealed in Petri dishes, particles were counted and assessed with Raman spectroscopy the same way as fishmeal samples. Petri dishes were never opened during visual inspection for enumeration; lids were only removed after enumeration was complete for transfer of particles for spectroscopy.

## Supplementary Information

Supplementary Information.

## Data Availability

Data supporting this study are openly available from the University of Southampton repository at https://doi.org/10.5258/SOTON/D1400.
