# Supplementary material for: Microplastics in fish and fishmeal: an emerging environmental challenge?
Source: Sci Rep. 2021 Jan 21;11:2045. doi: 10.1038/s41598-021-81499-8 (PMC7820289; doi:10.1038/s41598-021-81499-8)
Supplement: Supplementary file 1 — Supplementary Information. [file 41598_2021_81499_MOESM1_ESM.docx]

## Supplementary information

# Microplastics in fish and fishmeal: an emerging environmental challenge?

*Thiele, Christina J.^1^*; Hudson, Malcolm D.^1^; Russell, Andrea E.^2^; Saluveer, Marilin^1,3^; Sidaoui-Haddad, Giovanna^1^*

*^1^Centre for Environmental Science, Faculty of Environmental and Life Sciences, University of Southampton, University Road, Southampton SO17 1BJ, UK*

*^2^School of Chemistry, Faculty of Engineering and Physical Sciences, University of Southampton, SO17 1BJ, UK*

*^3^currently at Centre for Environmental Policy, Imperial College London, London, SW7 1NW, UK*

*corresponding author: Centre for Environmental Science, Faculty of Environmental and Life Sciences, University of Southampton, University Road, Southampton SO17 1BJ, UK; c.j.thiele@soton.ac.uk

Content of this file:

- Table S1 - Overview of methods from studies included in baseline review of microplastics in fishmeal-relevant fish species (page 2)
- Table S2 - Overview of microplastics in fishmeal-relevant fish species that are destined whole to fishmeal production (page 8)
- Table S3 - Overview of microplastics in fishmeal-relevant fish species of which mainly wastes and by-products are destined for fishmeal (page 12)
- Figure S1 – Set-up of overflow technique used for density separation
- Supplementary Note - Quantification of theoretical microplastic concentrations in fishmeal (page 26), including Table S4 for body weights of species
- Table S5- Recovery rates of dosed microplastics from fishmeal as totals and per polymer type (page 27)
- Supplementary Note - Quantification of fishmeal as microplastics pathway to the sea (page 28)
- Table S6- List of species used in fishmeal production
- References used in Supplementary Information

Supplementary Table S1 – Overview of methods from studies included in baseline review of microplastics in fishmeal-relevant fish species (n = 29), literature search for review performed 18^th^ March 2020. GIT = gastrointestinal tract; n/r = not reported. (Summarised headers provided on each page.)

[continues next page]

| Source | Study ID | Assessed body parts | Extraction method | ID method and percentage of particles assessed | Minimum score for confirmation and percentage of confirmed plastic | | Potential issues with quality & contamination control | Clean environment | Airborne controls | Additional notes |
| --- | --- | --- | --- | --- | --- | --- | --- | --- | --- | --- |
| Huang *et al.* 2020^1^ | 1 | Gills, GIT, liver, muscle (5g sub- sample) | 10% KOH digestion followed by NaCl density separation | FTIR; > 40% | n/r | n/r | No information on material of researchers' clothing, only dissections performed in clean fume hood, no airborne contamination controls | Dissections in clean fume hood, everything covered as much as possible | No | Only 5 g of muscle tissue (some fish weighed over 50g, heaviest 652.78g) |
| Zhang *et al*. 2020^2^ | 2 | GIT | 0.05M ferrous sulfate solution and 30% H_2_O_2_ digestion, followed by NaCl flotation | FTIR; 100% | 70% | n/r | Cotton laboratory coats worn, but no information on other materials worn by researchers, no airborne contamination controls | Clean fume hood | No |  |
| de Vries *et al.* 2020^3^ | 3 | GIT | 10% KOH digestion, and addition of citric acid | FTIR; < 14% were large enough for FTIR | 60% | n/r |  | No, but everything covered as much as possible and airborne contamination controls | Yes |  |
|  |  |  |  |  |  |  |  |  |  |  |
| Source | Study ID | Body parts | Extraction method | ID & assessed % of particles | Min. score & % confirmed | | Quality & contamination control potential issues | Clean environment | Airborne controls | Additional notes |
| Lopes *et al.* 2020^4^ | 4 | Stomach | 10 % KOH digestion | FTIR; 20% | 70% | n/r | Materials worn by researchers not mentioned | Clean air laminar flow hood, when not, then covered as much as possible, airborne contamination controls for microscope work | Partly | Airborne controls for microscope work |
| Kazour *et al.* 2019^5^ | 5 | GIT | 10% KOH digestion | Raman; 50% of filters, the ones with the heaviest potential microplastic load | n/r | n/r | No information of materials worn by researchers except lab coats; vague information about controls | Laminar flow hood and everything covered as much as possible | Unclear |  |
| Hossain *et al.* 2019^6^ | 6 | GIT | 30% H2O2 digestion followed by NaCl density separation | FTIR; 20% | n/r | 90% | While all clothing is cotton, face masks and hair nets (look synthetic) were worn; work performed in general lab space | No, but everything covered as much as possible | No | Five specimens pooled per samples, samples left to stand in NaCl for at least 2 (up to 5 or 6) weeks |
| Karbalaei *et al.* 2019^7^ | 7 | GIT, gills, kidney, liver, heart and spleen | 10% KOH digestion followed by density separation | Raman; 100% | n/r | 77% | While empty sample bags were used as airborne controls and did not reveal contamination, sample matrix inside might have led to microplastics coming off the polyethylene bags? | Laminar flow cabinet and everything covered as much as possible | Partly  [continues next page] | Airborne controls for dissections |
| Source | Study ID | Body parts | Extraction method | ID & assessed % of particles | Min. score & % confirmed | | Quality & contamination control potential issues | Clean environment | Airborne controls | Additional notes |
| Zhang *et al.* 2019^8^ | 8 | GIT and gills | 10% KOH digestion | FTIR; 100%? | 70% | n/r | No information on material of researchers' clothing, no airborne contamination controls | Laminar flow cabinet and everything covered as much as possible | No |  |
| Ding *et al*. 2019^9^ | 9 | GIT and gills | Gills: NaCl density separation; GIT 10% KOH digestion | FTIR; 100%? | 70% | n/r | limited information on clean environment, no information on material of researchers' clothing underneath cotton lab coats, no airborne contamination controls | "Semiclosed space" (not elaborated further) and everything covered as much as possible | No |  |
| Sun *et al.* 2019^10^ | 10 | GIT | 100% HNO3 digestion | FTIR; 46% | n/r | n/r | No information on contamination control provided | Unknown | No |  |
| Giani *et al.* 2019^11^ | 11 | GIT | 10% KOH digestion | FTIR; 100% | n/r | n/r | No information on contamination control provided | Unknown | No |  |
| McGoran *et al.* 2018^12^ | 12 | GIT | None | FTIR; 77% | n/r | 43% | No information on material of researchers' clothing | Everything covered as much as possible | Yes |  |
|  |  |  |  |  |  |  |  |  | [continues next page] |  |
|  |  |  |  |  |  |  |  |  |  |  |
| Source | Study ID | Body parts | Extraction method | ID & assessed % of particles | Min. score & % confirmed | | Quality & contamination control potential issues | Clean environment | Airborne controls | Additional notes |
| Bour *et al.* 2018^13^ | 13 | GIT | KOH digestion followed by NaCl density separation | FTIR; 100%? | 70% deemed positive, but scores ≥ 60% also visually checked | n/r | Not all their reagents were filtered | Clean room | No | Excluded 'fibres' (not regular and frayed ends) because found up to 30 per procedural blank, but included what they defined as 'lines' (regular shape, no frayed ends) |
| Markic *et al.* 2018^14^ | 14 | GIT | 15% H2O2 digestion | FTIR; 23% | 70% deemed positive, but scores ≥ 60% also visually checked | n/r | No information given on researcher's clothing | No | Yes | FTIR did not seem to have been used for adjusting potential microplastic counts, but rather to provide list of polymers found. |
| Digka *et al.* 2018^15^ | 15 | GIT | 30% H_2_O_2_ digestion | FTIR; 20% | 80% | n/r | Initial examination of GIT contents was performed before digestion, no information given on researcher's clothing | Use plastic cover for initial inspection, glove bag for rinsing and filtering, cover everything as much as possible | No |  |
| Cheung, Lui and Fok 2018^16^ | 16 | GIT | 30% H_2_O_2_ digestion followed by NaCl density separation | FTIR; 60% | n/r | 94% | No information on material of researcher's clothing | Everything covered as much as possible | Yes  [continues next page] |  |
| Source | Study ID | Body parts | Extraction method | ID & assessed % of particles | Min. score & % confirmed | | Quality & contamination control potential issues | Clean environment | Airborne controls | Additional notes |
| Halstead *et al.* 2018^17^ | 17 | GIT | None | FTIR and Raman; 69% | n/r | 55% for mullet, 69% for bream | Not much detail on contamination control | No | Yes | Results include natural fibres |
| Compa *et al.* 2018^18^ | 18 | GIT | None | FTIR; 100% | 70% | 59% | Not much detail on contamination control, e.g. researcher's clothing beyond lab coat | No | No | Results include natural fibres |
| Bessa *et al.* 2018^19^ | 19 | GIT | 10% KOH digestion | FTIR; 20% | 85% | n/r | Performed a 10-minute visual assessment prior to digestion, no information on researcher's clothing beyond lab coat | No (but restricted lab access) | Yes |  |
| Hermsen *et al.* 2017^20^ | 20 | Stomach | 10% KOH digestion | FTIR; 100% | n/r | n/r |  | Laminar flow cabinet | Partly | Airborne controls for microscope work |
| Karlsson *et al.* 2017^21^ | 21 | GIT | Proteinase K followed by 30% H_2_O_2_ digestion | FTIR; 10% | n/r | n/r | Limited information on contamination control, including materials of researcher's clothing | Everything covered as much as possible | No |  |
| Murphy *et al.* 2017^22^ | 22 | GIT | None | FTIR; 100% | n/r | n/r | Airborne control consisted of dry filter papers? | No | Yes |  |
|  |  |  |  |  |  |  |  |  | [continues next page] |  |
| Source | Study ID | Body parts | Extraction method | ID & assessed % of particles | Min. score & % confirmed | | Quality & contamination control potential issues | Clean environment | Airborne controls | Additional notes |
| Ory *et al.* 2017^23^ | 23 | GIT | None | FTIR; 36% | 85% | 93% | GIT stored in 95% ethanol before analysis, limited information on contamination control, e.g. researcher's clothing | No, but excluded fibres ≤0.05 mm in diameter | Partly | Airborne controls for microscope work |
| Tanaka and Takada 2016^24^ | 24 | GIT | 10% KOH digestion | FTIR; 100% | 70% | 87% | Limited information on contamination control, e.g. no information given on researcher's clothing | Everything covered as much as possible | Yes |  |
| Cannon *et al.* 2016^25^ | 25 | GIT | None | FTIR; 100% | n/r | n/r | Limited information on contamination control, e.g. no information given on researcher's clothing | Laminar flow cabinet (dissections) | No |  |
| Rummel *et al.* 2016^26^ | 26 | GIT | None | FTIR; 100% | n/r | 44% | No information on materials of researcher's clothing beyond lab coats | Everything covered as much as possible | Ambient air samples |  |
| Neves *et al.* 2015^27^ | 27 | Stomach | None | FTIR; 35% | n/r | 90% | Limited information on contamination control | Everything covered as much as possible | No |  |
|  |  |  |  |  |  |  |  |  |  |  |
|  |  |  |  |  |  |  |  |  |  |  |
|  |  |  |  |  |  |  |  |  |  |  |
|  |  |  |  |  |  |  |  |  | [continues next page] |  |
| Source | Study ID | Body parts | Extraction method | ID & assessed % of particles | Min. score & % confirmed | | Quality & contamination control potential issues | Clean environment | Airborne controls | Additional notes |
| Collard *et al.* 2015^28^ | 28 | Stomach | 9% NaCIO and 65% HNO3 digestion | Raman; 100% | n/r | 31% | Numerous steps before digestion (stomach removed, placed in formaldehyde, dissection, initial inspection, stomach contents placed in formaldehyde again, filtered for digestion), numerous steps needed in their digestion protocol, no information on researcher's clothing etc | Air flow hood (isolation of particles) | No |  |
| Lusher *et al.* 2013^29^ | 29 | GIT | None | FTIR; 100% | 70% deemed positive, but scores ≥ 60% also visually checked | n/r | Limited information on contamination control, e.g. no information given on researcher's clothing | No | No | Inspection limited to 10 minutes. Unknown number of juveniles included. |

Supplementary Table S2 – Overview of microplastics in fishmeal-relevant fish species that are destined whole to fishmeal production – concentrations, sizes and additional information based on a baseline review (literature search conducted 18^th^ March 2020). When contamination control measures of a study indicated that fibre contamination could have been an issue, concentrations were adjusted in the column “adjusted concentrations” by calculating abundance and total number based on percentages of fibres reported by individual studies. “Overall” refers to overall study results when results were not reported separately by species. Abbreviations used: frag. = fragments, Med. Sea = Mediterranean Sea, n/r = not reported; PS = polystyrene, PP = polypropylene, PE = polyethylene, PA = nylon, PET = polyethylene terephthalate, PUR = polyurethane, ABS = acrylonitrile butadiene styrene, PMMA = polymethyl methacrylate. (Summarised headers provided on each page.)

[continues next page]

|  |  |  |  | Microplastics concentrations | | | Adjusted concentrations | | Size information | | Additional information | | |  |  |
| --- | --- | --- | --- | --- | --- | --- | --- | --- | --- | --- | --- | --- | --- | --- | --- |
| Species and location |  | n | Mean weight (g) | % with micro- plastic occurrence | Mean abundance per specimen | Total number found in species | Abundance non-fibrous per specimen | Total number non-fibrous in species | Filter size, Limit of detection (LOD) of method used | Minimum/mean/ median/ range of particle size | Polymer | Category | Colour | Additional notes | Study ID |
| *Ammodytes personatus* | Yellow Sea | 50 | 7 | 40 | 0.54 (all fish), 1.4 (fish with plastic) | 3 not fibre | 0.06 (all fish), 0.15 (fish with plastic) | 3 | 0.45 µm filter | Overall: 16-4,740 µm, mean 941 µm, about 22% 16-200 µm |  | 89% fibres, 11% pellets, 0% frag. |  |  | 10 |
| *Engraulis encrasicolus* | Med. Sea (east) | 30 | n/r | 83.4 | 2.5 |  | 1.4 |  | 1.6 µm filter | Most items < 200 µm, followed by 200-400 µm | PS followed by PP, PE, PA, PET, PUR and ABS | 1.3 frag. per fish, 1.1 fibres and 0.1 films | Frag. mainly blue, fibres mainly black |  | 5 |
| *Engraulis encrasicolus* | Med. Sea (west) | 3 | n/r | n/r | Overall: 1.2 (incl. > 5,000 µm) | Overall: 11 (incl. > 5,000 µm) | Not possible | Not possible | 5 µm filter, but dried under 36 µm mesh | 220-9,490 µm, mean 2,080 µm | Overall: PE, PP and PET | n/r | Overall: clear, white, blue & green |  | 28 |
|  |  |  |  |  |  |  |  |  |  |  |  |  |  |  |  |
|  |  |  | Mean | Microplastics concentrations | | | Adj. concentrations | | Size information | | Additional information | | |  |  |
| Species and location | | n | weight (g) | % FO | Abundance/ specimen | Totals/ species | Abundance non-fib. | Totals non-fib. | Filter size & LOD | Particle size info | Polymer | Category | Colour | Add. notes | Study ID |
| *Engraulis encrasicolus* | Med. Sea (west) | 105 | 13 | 14.28 includes natural fibres | 0.18 (incl. natural fibres) | Overall: 41 | 0.039 |  | Unknown* | n/r | Overall: 30% PET | Overall (incl. natural fibres): 83% fibres | Overall: mainly blue and white | Adjusted based on finding that only 21.4% were plastic | 18 |
| *Engraulis encrasicolus* | North East Atlantic | 131 | n/r | 79 | 0.48 (median) | 77 |  |  | 20 µm filter | Overall: 20-5,000 µm |  | Overall: 80% fibres | Overall: mainly blue and black |  | 4 |
| *Engraulis japonicus* | North Pacific Ocean | 64 | n/r | 77 | 2.3 (max 15 items per specimen) |  |  |  | Unknown | 80% were 150-1000 µm | PE and PP | 86% frag., 7.3% beads |  |  | 24 |
| *Engraulis japonicus* | Yellow Sea | 280 | 4 | 32.9 | 0.39 per all, 1.2 mps per fish with plastic | 47 not fibre | 0.17 (all fish), 0.52 (fish with plastic) | 47 | 0.45 µm | Overall: 16-4,740 µm, mean 941 µm, about 21-22% of particles between 16-200 µm |  | 57% fibres, 21% pellets, 22% frag. |  |  | 10 |
|  |  |  |  |  |  |  |  |  |  |  |  |  |  | [continues next page] |  |
|  |  |  |  |  |  |  |  |  |  |  |  |  |  |  |  |
|  |  |  | Mean | Microplastics concentrations | | | Adj. concentrations | | Size information | | Additional information | | |  |  |
| Species and location | | n | weight (g) | % FO | Abundance/ specimen | Totals/ species | Abundance non-fib. | Totals non-fib. | Filter size & LOD | Particle size info | Polymer | Category | Colour | Add. notes | Study ID |
| *Euthynnus affinis* | Malaysia (fish- market) | 10 | 187 | 0 |  | 0 |  |  | 149 µm | Overall: minimum 215 µm | Overall: mainly PE | Overall: 67.4% frag., 16.3% fibre, 16.3% film |  | Body parts pooled | 7 |
| *Micromesistius poutassou* | North East Atlantic | 27 | n/r | 52% | c.2 | 29 | 0.63 | 9 |  | Overall: 130-14,300 µm, 94.4% of particles were microplastics (i.e. < 5,000 µm), 31% < 1,000 µm | 13 rayon, 16 PA, 1 poly- ester | Overall: 68.3% fibres, 16.1% frag., 11.5% beads | Overall: 45.4% black | Based on polymer types, could most particles be fibres? | 29 |
| *Micromesistius poutassou* | North East Atlantic | 20 | n/r | 0 | 0.0 | 0 | 0 | 0 | Unknown* | 100-15,000 µm, 75% < 5,000 µm, 35% of particles 100-1,0000 µm | Overall: PA, followed by PET, acrylic, PP and PS and PET/PP mix | Overall: 82.1% fibres |  | Species of non-interest did contain micro- plastics | 22 |
| *Sprattus sprattus* | North Sea | 141 | n/r | 0.71 | 2 | 2 |  |  | 20 µm sieve |  | PMMA | Spherical particles |  |  | 20 |
|  |  |  |  |  | | |  | |  | |  | | |  |  |
|  |  |  |  |  | | |  | |  | |  | | |  |  |
|  |  |  |  |  | | |  | |  | |  | | | [continues next page] |  |
|  |  |  | Mean | Microplastics concentrations | | | Adj. concentrations | | Size information | | Additional information | | |  |  |
| Species and location | | n | weight (g) | % FO | Abundance/ specimen | Totals/ species | Abundance non-fib. | Totals non-fib. | Filter size & LOD | Particle size info | Polymer | Category | Colour | Add. notes | Study ID |
| *Trachurus trachurus* | North East Atlantic | 56 | n/r | 29% | c.1.5 | 24 | 0.48 | 8 |  | Overall: 130-14,300 µm, 94.4% of particles were microplastics (i.e. < 5,000 µm), 31% < 1,000 µm | 12 rayon, 1 acrylic, 1 poly- ester | Overall: 68.3% fibres, 16.1% frag., 11.5% beads | Overall: 45.4% black | Based on polymer types, could most particles be fibres? | 29 |
| *Trachurus trachurus* | North East Atlantic | 24 | n/r | 100 | 1.75 (median) | 41 |  |  | 20 µm filter | Overall: 20-5,000 µm |  | Overall: 80% fibres | Overall: mainly blue and black |  | 4 |
| *Trachurus trachurus* | North East Atlantic | 5 | n/r | 0 | 0.0 | 0 | 0 | 0 | Unknown* | 100-15,000 µm, 75% < 5,000 µm, 35% of particles 100-1,0000 µm | Overall: PA, followed by PET, acrylic, PP and PS and PET/PP mix | Overall: 82.1% fibres |  | Species of non-interest did contain microplastics | 22 |
|  |  |  |  |  |  |  |  |  |  |  |  |  |  | [continues next page] |  |
|  |  |  | Mean | Microplastics concentrations | | | Adj. concentrations | | Size information | | Additional information | | |  |  |
| Species and location | | n | weight (g) | % FO | Abundance/ specimen | Totals/ species | Abundance non-fibrous | Totals non-fib. | Filter size & LOD | Particle size info | Polymer | Category | Colour | Add. notes | Study ID |
| *Trachurus trachurus* | North East Atlantic | 44 | 153 | 6.8 | 0.07 | 3 | 0.02 | 1 | Unknown* | 217-4,810 µm, mean 2,110 µm | Overall: PP, PE, alkyd resin, rayon, poly- ester, PA and acrylic | Overall: 65.8% fibres, 34.2% frag. |  |  | 27 |
| *Trisopterus esmarki* | North Sea | 20 | n/r | 25 | n/r | n/r |  |  | 8 µm filter | Overall: 41-9,000 (line length) µm, 12% <100 µm, 47% 100-200 µm | All PE | 100% fibres (lines according to their definition) | Overall: mainly blue and clear |  | 13 |

Supplementary Table S3 – Overview of microplastics in fishmeal-relevant fish species of which mainly wastes and by-products are destined for fishmeal – concentrations, sizes and additional information based on a baseline review (literature search conducted 18^th^ March 2020). When contamination control measures of a study indicated that fibre contamination could have been an issue, concentrations were adjusted in the column “adjusted concentrations” by calculating abundance and total number based on percentages of fibres reported by individual studies. “Overall” refers to overall study results when results were not reported separately by species. Abbreviations used: frag. = fragments, Med. Sea = Mediterranean Sea, n/r = not reported; PS = polystyrene, PP = polypropylene, PE = polyethylene, PA = nylon, PET = polyethylene terephthalate, PUR = polyurethane, ABS = acrylonitrile butadiene styrene, PMMA = polymethyl methacrylate. (Summarised headers provided on each page.)

[continues next page]

|  |  |  | Microplastics concentrations | | | Adjusted concentrations | | Size information | | Additional information | | |  |  |
| --- | --- | --- | --- | --- | --- | --- | --- | --- | --- | --- | --- | --- | --- | --- |
| Species and location | n | Mean weight (g) | % with micro- plastic occurrence | Mean abundance per specimen | Total number found in species | Abundance non-fibrous per specimen | Total number non-fibrous in species | Filter size, Limit of detection (LOD) of method used | Minimum/mean/ median/ range of particle size | Polymer | Category | Colour | Additional notes | Study ID |
| *Anguilla anguilla* North Sea | 2 | n/r | 50 | 2 | Overall: > 3000 |  |  | Unknown* | n/r |  |  |  |  | 12 |
| *Argyrosomus regius*, Portugal (fish market) | 5 | n/r | 60.0 | 0.8 | 4 | 0.27 | 1.4 | Unknown* | 217-4,810 µm, mean 2,110 µm | Overall: PP, PE, alkyd resin, rayon, poly- ester, nylon and acrylic | Overall: 65.8% fibres, 34.2% frag. |  |  | 27 |
| *Chrysochir aureus*, East China Sea | 30 | 40 | Overall: 57.5% | c. 0.3 gill and GIT each |  | 0.12 (in both tissues) |  | 1.6 µm | Overall: 24.6-268.0 µm in gills, 32.9-4092.2 µm in GIT, overall mean 727 µm | Overall: mainly PET, followed by PE and others | Overall: 59.6% fibres, 38% frag., 9.3% film 2.3% foam, 1.7% pellets | Overall: mainly blue, black then red |  | 8 |
|  |  | Mean | Microplastics concentrations | | | Adj. concentrations | | Size information | | Additional information | | |  |  |
|  | n | weight (g) | % FO | Abundance/ specimen | Totals/ species | Abundance non-fib. | Totals non-fib. | Filter size & LOD | Particle size info | Polymer | Category | Colour | Add. notes | Study ID |
| *Clupea harengus*, North and Baltic Seas | 33 | 75 | 0 | 0 | 0 |  |  | 500 µm sieve (but included smaller particles when found) | Overall: minimum 180 µm |  |  |  | Items >5,000 µm excluded here; species of non-interest did contain micro- plastics | 26 |
| *Clupea harengus*, North Sea | 3 | n/r | n/r | Overall: 1.2 (incl. > 5,000 µm) | Overall: 11 (incl. > 5,000 µm) | Not possible | Not possible | 5 µm filter, but dried under 36 µm mesh | 220-9,490 µm, mean 2,080 µm | Overall study result: PE, PP and PET | n/r | Overall: clear, white, blue and green |  | 28 |
| *Clupea harengus*, North Sea | 131 | n/r | 0 |  |  |  |  | 20 µm sieve |  |  |  |  |  | 20 |
| *Collichthys lucidus*, East China Sea | 3 | n/r |  | 6 |  |  |  | LOD for FTIR 50 µm |  | Overall: cello- phane, PA, PE and PP | Overall: 83.5% fibres | Overall: mainly blue |  | 2 |
| *Collichthys lucidus*, East China Sea | 5 | 124 | Overall: 57.5% | None in gills, c.1.2 mps in GIT | 6 in GIT overall | 0.48 in GIT | 2.42 | 1.6 µm | Overall: 24.6-268.0 µm in gills, 32.9-4092.2 µm in GIT, overall mean 727 µm | Overall: mainly PET, followed by PE and others | Overall: 59.6% fibres, 38% frag., 9.3% film 2.3% foam, 1.7% pellets | Overall: mainly blue, black then red |  | 8 |
|  |  |  |  |  |  |  |  |  |  |  |  |  | [continues next page] |  |
|  |  | Mean | Microplastics concentrations | | | Adj. concentrations | | Size information | | Additional information | | |  |  |
|  | n | weight (g) | % FO | Abundance/ specimen | Totals/ species | Abundance non-fib. | Totals non-fib. | Filter size & LOD | Particle size info | Polymer | Category | Colour | Add. notes | Study ID |
| *Collichthys lucidus*, East China Sea | 5 | 124 | Overall: 57.5% | None in gills, c.1.2 mps in GIT | 6 in GIT overall | 0.48 in GIT | 2.42 | 1.6 µm | Overall: 24.6-268.0 µm in gills, 32.9-4092.2 µm in GIT, overall mean 727 µm | Overall: mainly PET, followed by PE and others | Overall: 59.6% fibres, 38% frag., 9.3% film 2.3% foam, 1.7% pellets | Overall: mainly blue, black then red |  | 8 |
| *Collichthys niveatus*, East China Sea | 1 | 154 | Overall: 57.5% | None in gills or GIT | 0 | 0 | 0 | 1.6 µm | Overall: 24.6-268.0 µm in gills, 32.9-4092.2 µm in GIT, overall mean 727 µm | Overall: mainly PET, followed by PE and others | Overall: 59.6% fibres, 38% frag., 9.3% film 2.3% foam, 1.7% pellets | Overall: mainly blue, black then red |  | 8 |
| *Decapterus macrosoma*, South Pacific Ocean | 25 | 68 | 28 | 1.1 | 8 |  |  | Smallest filter used 63 µm | Overall: 1% < 100 µm, 38% 100-500 µm, 24% 500-1000 µm | Overall: mainly poly- ester, PE and rayon | Overall: 49% frag., 33% fibres, 18% film | Overall: black, blue, white, clear |  | 14 |
| *Decapterus maruadsi*, Yellow Sea | 78 | 17 | 30.8 | 0.41 per all, 1.3 mps per fish with plastic | 6 not fibre | 0.07 (all fish), 0.23 (fish with plastic) | 6 | 0.45 µm | Overall: 16-4,740 µm, mean 941 µm, about 21-22% of particles between 16-200 µm |  | 81% fibres, 9% pellets, 9% frag. | [continues next page] |  | 10 |
|  |  | Mean | Microplastics concentrations | | | Adj. concentrations | | Size information | | Additional information | | |  |  |
|  | n | weight (g) | % FO | Abundance/ specimen | Totals/ species | Abundance non-fib. | Totals non-fib. | Filter size & LOD | Particle size info | Polymer | Category | Colour | Add. notes | Study ID |
| *Decapterus muroadsi*, South Pacific Ocean | 25 | 119 | 64 | 2.4 | 39 |  |  | Smallest filter used 63 µm | Overall: 1% < 100 µm, 38% 100-500 µm, 24% 500-1000 µm | Overall: mainly poly- ester, PE and rayon | Overall: 49% frag., 33% fibres, 18% film | Overall: black, blue, white, clear |  | 14 |
| *Decapterus muroadsi*, South Pacific Ocean | 20 | n/r | 80 | 2.5 overall, 3.1 for only fish that had mps (max 5 items) | 20 |  |  | Unknown* | 200-5,000 µm, median 1.3 mm, 8% <300 µm, c.31% < 1,000 µm | overall (incl. water samples): mainly PE | 92% frag. (hard and soft), 6% threads, 2% films | mainly blue |  | 23 |
| *Dendrophysa russelii*, South China Sea | 2 | 18 |  | 5 (one 8, one 2) | 10 | 1.5 | 3 | 20 µm filter | Overall: 20-5,000µm, with 46% 20-1,000µm | Overall: PE, PET, PP, Cello- phane, PS, PA and PUR | Overall: 70% fibres, 18% frag., 9% film, 3% pellets | Overall: black, blue, red, green and others |  | 1 |
| *Dicentrarchus labrax*, North East Atlantic | 40 | 50 | 23 | 0.3 | Overall: 157 | 0.01 |  | Filter 1.2 µm | c.15% particles < 1,000 µm | Overall: mainly poly- ester, rayon, PP, PAN | Overall: 96% fibres, 4% frag. | Blue, followed by clear |  | 19 |
| *Eupleurogramms muticus*, Yellow Sea | 15 | 16 | 33.3 | 0.33 (all fish) and 1.0 (fish with plastic) | 1 not fibre | 0.07 (all fish), 0.20 (fish with plastic) | 1 | 0.45 µm | Overall: 16-4,740 µm, mean 941 µm, about 21-22% of particles between 16-200 µm |  | 80% fibres, 20% pellets, 0% frag. | [continues next page] |  | 10 |
|  |  | Mean | Microplastics concentrations | | | Adj. concentrations | | Size information | | Additional information | | |  |  |
|  | n | weight (g) | % FO | Abundance/ specimen | Totals/ species | Abundance non-fib. | Totals non-fib. | Filter size & LOD | Particle size info | Polymer | Category | Colour | Add. notes | Study ID |
| *Gadus macrocephalus*, Yellow Sea | 40 | 13 | 40 | 0.43 (all fish), 1.2 (fish with plastic) | 6 not fibre | 0.15 (all fish), 0.42 (fish with plastic) | 6 | 0.45 µm | Overall: 16-4,740 µm, mean 941 µm, about 21-22% of particles between 16-200 µm |  | 65% fibres, 29% pellets, 6% frag. |  |  | 10 |
| *Gadus morhua*, North and Baltic Seas | 81 | 413 | 0 | 0 | 0 |  |  | 500 µm sieve (but if found included smaller particles) | Overall: minimum 180 µm |  |  |  | Items >5,000 µm excluded here; species of non-interest did contain micro- plastics | 26 |
| *Gadus morhua*, North Atlantic Ocean | 39 | 6090 | 20.5 | 0.23 | 9 |  |  | LOD for FTIR given as 80 µm |  | PP and PE (one each) | 5 fibres, 4 frag. | 34% blue, 33% green |  | 3 |
| *Gadus morhua*, North East Atlantic | 2 | n/r | 0 | 0 | Overall: > 3000 |  |  | Unknown* | n/r |  |  |  |  | 12 |
| *Gadus morhua*, North Sea | 1 | n/r | 0 | 0 | Overall: > 3000 |  |  | Unknown* | n/r |  |  |  |  | 12 |
| *Gnathodentex aureolineatus*, South China Sea | 1 | 50 | 100 | 1 in gill, 0 in GIT |  | 0.35 (gills), 0 (GIT) |  | LOD 6.25 µm | Overall: mainly 20-330 µm, 3.7% < 20 µm, range 10-3,008 µm, mean 687.5 µm |  | Overall: c. 65% fibres, 15% frag., 20% granules | Overall: mainly black, blue and white | Fibres mainly account for particles >330 µm | 9 |
| *Gnathodentex aureolineatus*, South Pacific Ocean | 29 | 263 | 6.9 | 1 | 2 |  |  | Smallest filter used 63 µm | Overall: 1% < 100 µm, 38% 100-500 µm, 24% 500-1000 µm | Overall: mainly poly- ester, PE and rayon | Overall: 49% frag., 33% fibres, 18% film | Overall: black, blue, white, clear  [continues next page] |  | 14 |
|  |  | Mean | Microplastics concentrations | | | Adj. concentrations | | Size information | | Additional information | | |  |  |
|  | n | weight (g) | % FO | Abundance/ specimen | Totals/ species | Abundance non-fib. | Totals non-fib. | Filter size & LOD | Particle size info | Polymer | Category | Colour | Add. notes | Study ID |
| *Johnius* spp., East China Sea | 30 | 39 | Overall: 57.5% | c.0.4 mps/gill and 0.5 mps/GIT |  | 0.16 (gills), 0.20 (GIT) |  | 1.6 µm | Overall: 24.6-268.0 µm in gills, 32.9-4092.2 µm in GIT, overall mean 727 µm | Overall: mainly PET, followed by PE and others | Overall: 59.6% fibres, 38% frag., 9.3% film 2.3% foam, 1.7% pellets | Overall: mainly blue, black then red |  | 8 |
| *Katsuwonus pelamis*, South Pacific Ocean | 26 | 6018 | 23.1 | 1.5 | 9 |  |  | Smallest filter used 63 µm | Overall: 1% <100 µm, 38% 100-500 µm, 24% 500-1000 µm | Overall: mainly poly- ester, PE and rayon | Overall: 49% frag., 33% fibres, 18% film | Overall: black, blue, white, clear |  | 14 |
| *Katsuwonus pelamis*, Tasman Sea | 1 | 1629 | 0 |  |  | 0 |  | Unknown* |  |  |  |  | Only found microplastics in 1 of 21 species analysed | 25 |
| *Larimichthys crocea*, East China Sea | 3 | n/r |  | 5.5 |  |  |  | LOD for FTIR was 50 µm |  | Overall: cello- phane, PA, PE and PP | Overall: 83.5% fibres | Overall: mainly blue |  | 2 |
| *Larimichthys crocea*, East China Sea | 30 | 37 | Overall: 57.5% | c.0.2 (gills); 0.70 (GIT) |  | 0.08 (gills), 0.28 (GIT) |  | 1.6 µm | Overall: 24.6-268.0 µm in gills, 32.9-4092.2 µm in GIT, overall mean 727 µm | Overall: mainly PET, followed by PE and others | Overall: 59.6% fibres, 38% frag., 9.3% film 2.3% foam, 1.7% pellets | Overall: mainly blue, black then red  [continues next page] |  | 8 |
|  |  | Mean | Microplastics concentrations | | | Adj. concentrations | | Size information | | Additional information | | |  |  |
|  | n | weight (g) | % FO | Abundance/ specimen | Totals/ species | Abundance non-fib. | Totals non-fib. | Filter size & LOD | Particle size info | Polymer | Category | Colour | Add. notes | Study ID |
| *Larimichthys polyactis*, Yellow Sea | 30 | 22 | 36.7 | Highest overall 0.97 mps for all, 2.64 for the ones with plastic only | 18 not fibre | 0.60 (all fish), 1.64 (fish with plastic) | 18 | 0.45 µm | Overall: 16-4,740 µm, mean 941 µm, about 21-22% of particles between 16-200 µm |  | 38% fibres, 62% pellets, 0% frag. |  |  | 10 |
| *Lethrinus amboinensis*, South Pacific Ocean | 26 | 656 | 23.1 | 1.7 | 10 |  |  | Smallest filter used 63 µm | Overall: 1% < 100 µm, 38% 100-500 µm, 24% 500-1000 µm | Overall: mainly poly- ester, PE and rayon | Overall: 49% frag., 33% fibres, 18% film | Overall: black, blue, white, clear |  | 14 |
| *Lethrinus obsoletus*, South Pacific Ocean | 3 | 244 | 13.3 | 1.3 | 5 |  |  | Smallest filter used 63 µm | Overall: 1% < 100 µm, 38% 100-500 µm, 24% 500-1000 µm | Overall: mainly poly- ester, PE and rayon | Overall: 49% frag., 33% fibres, 18% film | Overall: black, blue, white, clear |  | 14 |
| *Melanogrammus aeglefinus*, North East Atlantic | 6 | n/r | 100 | 5.83 | Overall: >3000 |  |  | unknown* | n/r |  |  |  |  | 12 |
| *Merluccius merluccius*, central Med. Sea | 97 | n/r | 26.8 | 1.38, max 8 mps per specimen | 37 | 0.26, max. 1.52 per specimen | 7 | LOD 100 µm | 17% 100-300 µm, 11% 300-500 µm, 36% 500-1,000 µm |  | 81% fibres, 19% fragments |  | only analysed GITs that were full | 11 |
|  |  |  |  |  |  |  |  |  |  |  |  |  | [continues next page] |  |
|  |  | Mean | Microplastics concentrations | | | Adj. concentrations | | Size information | | Additional information | | |  |  |
|  | n | weight (g) | % FO | Abundance/ specimen | Totals/ species | Abundance non-fib. | Totals non-fib. | Filter size & LOD | Particle size info | Polymer | Category | Colour | Add. notes | Study ID |
| *Merluccius merluccius*, North East Atlantic | 7 | 344 | 28.6 | 0.29 | 2 | 0.099 | 0.7 | unknown* | 217-4,810 µm, mean 2,110 µm | Overall: PP, PE, alkyd resin, rayon, poly- ester, PA and acrylic | Overall: 65.8% fibres, 34.2% frag. |  |  | 27 |
| *Mugil cephalus*, South China Sea | 60 | 741 | 60 (wild) 16.7 (captive) | 4.3 (wild) 0.2 (captive) | 129 (80 in one specimen?), overall only 6 in captive |  |  | 11 µm filter | Minimum 0.1 mm, mean 1.18 mm (SD 0.767), median 1; 83-90% (captive/ wild) < 2mm | 42% PP, 25% PE | Captive: only fibres, wild: 60% fibres, 34% frag., 6% sheets | 44% green, 16% blue, 15% black | 30 captive and 30 wild fish | 16 |
| *Mugil cephalus*, South Pacific Ocean | 22 | n/a | 13.6 | 2 | 6 |  |  | Smallest filter used 63 µm | Overall: 1% < 100 µm, 38% 100-500 µm, 24% 500-1000 µm | Overall: mainly poly- ester, PE and rayon | Overall: 49% frag., 33% fibres, 18% film | Overall: black, blue, white, clear |  | 14 |
| *Mugil cephalus*, Tasman Sea | 45 | 228 |  | 2.5 | 60 |  |  | Unknown* | n/r |  | Overall: of 66, 63 fibres and 3 frag. |  |  | 17 |
| *Mugil cephalus*, Tasman Sea | 43 | 247 | 0 |  |  | 0 |  | Unknown* |  |  |  |  | Only found microplastics in 1 of 21 species analysed | 25 |
|  |  |  |  |  |  |  |  |  |  |  |  |  |  |  |
|  |  | Mean | Microplastics concentrations | | | Adj. concentrations | | Size information | | Additional information | | |  |  |
|  | n | weight (g) | % FO | Abundance/ specimen | Totals/ species | Abundance non-fib. | Totals non-fib. | Filter size & LOD | Particle size info | Polymer | Category | Colour | Add. notes | Study ID |
| *Muraenesox cinereus*, East China Sea | 3 | n/r |  | 7 |  |  |  | LOD for FTIR was 50 µm |  | Overall: cello- phane, PA, PE and PP | Overall: 83.5% fibres | Overall: mainly blue |  | 2 |
| *Muraenesox cinereus*, East China Sea | 30 | 145 | Overall: 57.5% | 1.93 (gills; 0-7 items), c.0.75 (GIT; 0-3 items) |  | 0.78 (gills; 0-2.82), 0.30 (GIT; 0-1.21) |  | 1.6 µm | Overall: 24.6-268.0 µm in gills, 32.9-4092.2 µm in GIT, overall mean 727 µm | Overall: mainly PET, followed by PE and others | Overall: 59.6% fibres, 38% frag., 9.3% film 2.3% foam, 1.7% pellets | Overall: mainly blue, black then red |  | 8 |
| *Muraenesox cinereus*, South China Sea | 1 | 653 | 100 | 3 | 3 | 2 | 2 |  | Overall: 20-5,000µm, with 46% 20-1,000µm | Overall: PE, PET, PP, cello- phane, PS, PA and PU | Overall: 70% fibres, 18% frag., 9% film, 3% pellets | Overall: black, blue, red, green and others |  | 1 |
| *Nemipterus bipunctatus*, Malaysia (fish market) | 10 | 88 | 10 |  | n/r |  |  | 149 µm | Overall: minimum 215 µm | Overall: mainly PE | Overall: 67.4% frag., 16.3% fibre, 16.3% film |  | body parts pooled | 7 |
| *Pampus argenteus*, Yellow Sea | 10 | 14 | 20 | Lowest overall 0.2 and 1 (all/plastic only specimens) | 0 not fibre | 0 | 0 | 0.45 µm | Overall: 16-4,740 µm, mean 941 µm, about 21-22% of particles between 16-200 µm |  | 100% fibres, 0% pellets, 0% frag. |  |  | 10 |
|  |  |  |  |  |  |  |  |  |  |  |  | [continues next page] |  |  |
|  |  | Mean | Microplastics concentrations | | | Adj. concentrations | | Size information | | Additional information | | |  |  |
|  | n | weight (g) | % FO | Abundance/ specimen | Totals/ species | Abundance non-fib. | Totals non-fib. | Filter size & LOD | Particle size info | Polymer | Category | Colour | Add. notes | Study ID |
| *Pennahia argentata*, East China Sea | 4 | 90 | Overall: 57.5% | 0.5 mps in gill, none in GIT | 2 mps in gill of one specimen | 0.20 (gills) | 0.81 in gills of one specimen | 1.6 µm | Overall: 24.6-268.0 µm in gills, 32.9-4092.2 µm in GIT, overall mean 727 µm | Overall: mainly PET, followed by PE and others | Overall: 59.6% fibres, 38% frag., 9.3% film 2.3% foam, 1.7% pellets | Overall: mainly blue, black then red |  | 8 |
| *Pennahia macroephalus*, East China Sea | 1 | 132 | Overall: 57.5% | None in gills or GIT | 0 | 0 | 0 | 1.6 µm | Overall: 24.6-268.0 µm in gills, 32.9-4092.2 µm in GIT, overall mean 727 µm | Overall: mainly PET, followed by PE and others | Overall: 59.6% fibres, 38% frag., 9.3% film 2.3% foam, 1.7% pellets | Overall: mainly blue, black then red |  | 8 |
| *Pollachius pollachius*, North East Atlantic | 5 | n/r | 0 | 0 | 0 | 0 | 0 | Unknown* | 100-15,000 µm, 75% < 5,000 µm, 35% of particles 100-1,0000 µm | Overall: PA, followed by PET, acrylic, PP and PS and PET/PP mix | Overall: 82.1% fibres |  | Species of non-interest did contain microplastics | 22 |
| *Pollachius virens*, North Atlantic Ocean | 46 | 1950 | 17.4 | 0.28 | 13 |  |  | LOD for FTIR given as 80 µm |  | 1 PP iden- tified | 8 fibres, 5 frag. | 38% blue, 23% black |  | 3 |
|  |  |  |  |  |  |  |  |  |  |  |  | [continues next page] |  |  |
|  |  | Mean | Microplastics concentrations | | | Adj. concentrations | | Size information | | Additional information | | |  |  |
|  | n | weight (g) | % FO | Abundance/ specimen | Totals/ species | Abundance non-fib. | Totals non-fib. | Filter size & LOD | Particle size info | Polymer | Category | Colour | Add. notes | Study ID |
| *Rastrelliger kanagurta*, Malaysia (fish market) | 10 | 110 | 50 |  | 5 |  |  | 149 µm | Overall: minimum 215 µm | Overall: mainly PE | Overall: 67.4% frag., 16.3% fibre, 16.3% film |  | Body parts pooled | 7 |
| *Salmo trutta*, North Sea | 62 | n/r | 68% |  | 122 | Not possible | Not possible | Filter 1.2 µm |  | PP, alkyd resin, PS and PMMA | n/r |  |  | 21 |
| *Sardina pilchardus*, central Med. Sea | 36 | 10 | 47.2 | 1.8, max 4 (2 fibre & 2 frag.) (0.8 across all specimens) | Overall: 125 | 1.44, max. 2 (0.64 across all specimens) |  | Filter 1.2 µm | 39-857 µm, overall: minimum 32 µm |  | 80% frag., 20% fibres | Overall (fish): blue and pink |  | 15 |
| *Sardina pilchardus*, western Med. Sea | 105 | 42 | 15.24 incl. natural fibres | 0.21 (incl. natural fibres) | Overall: 41 | 0.15 |  | Unknown* | n/r | Overall: 30% PET | Overall (incl. natural fibres): 83% fibres | Overall: mainly blue and white | Adjusted concentrations based on finding that 70% were plastic | 18 |
| *Sardina pilchardus*, North East Atlantic | 3 | n/r | n/r | Overall: 1.2 (incl. >5,000 µm) | Overall: 11 (incl. >5,000 µm) | Not possible | Not possible | 5 µm filter, but dried under 36 µm mesh | 220-9,490 µm, mean 2,080 µm | Overall: PE, PP and PET | n/r | Overall: clear, white, blue and green |  | 28 |
| *Sardina pilchardus*, North East Atlantic | 76 | n/r | 58 | 0.16 (median) | 22 |  |  | 20 µm filter | Overall: 20-5,000 µm |  | Overall: 80% fibres | Overall: mainly blue and black |  | 4 |
|  |  |  |  |  |  |  |  |  |  |  |  | [continues next page] |  |  |
|  |  | Mean | Microplastics concentrations | | | Adj. concentrations | | Size information | | Additional information | | |  |  |
|  | n | weight (g) | % FO | Abundance/ specimen | Totals/ species | Abundance non-fib. | Totals non-fib. | Filter size & LOD | Particle size info | Polymer | Category | Colour | Add. notes | Study ID |
| *Sardina pilchardus*, North East Atlantic | 12 | 77 | 0.0 | 0 | 0 | 0 | 0 | Unknown* | 217-4,810 µm, mean 2,110 µm | Overall: PP, PE, alkyd resin, rayon, poly- ester, nylon and acrylic | Overall: 65.8% fibres, 34.2% frag. |  |  | 27 |
| *Sardinella gibbosa*, Bay of Bengal | 25 | 38 |  | 3.2 | 80 (range 11-26) | 1.44 | 36 | 46 µm sieve | 43% < 500 µm, 37% 500-1,000 µm, 20% > 1,000 µm | Overall: mainly PA, followed by PET | 26% frag., 55% fibres, 19% particles | Mainly white (60%) and black (21%) |  | 6 |
| *Scomber australasicus*, Tasman Sea | 4 | 371 | 0 |  |  | 0 |  | Unknown* |  |  |  |  | Only found microplastics in 1 of 21 species analysed | 25 |
| *Scomber japonicus*, North East Atlantic | 35 | 228 | 31.4 | 0.57 | 20 | 0.19 | 6.8 | Unknown* | 217-4,810 µm, mean 2,110 µm | Overall: PP, PE, alkyd resin, rayon, poly- ester, nylon and acrylic | Overall: 65.8% fibres, 34.2% frag. |  |  | 27 |
| *Scomber scombrus*, North East Atlantic | 19 | n/r | 100 | 1 (median) | 17 |  |  | 20 µm filter | Overall: 20-5,000 µm |  | Overall: 80% fibres | Overall: mainly blue and black  [continues next page] |  | 4 |
|  |  | Mean | Microplastics concentrations | | | Adj. concentrations | | Size information | | Additional information | | |  |  |
|  | n | weight (g) | % FO | Abundance/ specimen | Totals/ species | Abundance non-fib. | Totals non-fib. | Filter size & LOD | Particle size info | Polymer | Category | Colour | Add. notes | Study ID |
| *Scomber scombrus*, North East Atlantic | 13 | 190 | 30.8 | 0.46 | 6 | 0.16 | 2.1 | Unknown* | 217-4,810 µm, mean 2,110 µm | Overall: PP, PE, alkyd resin, rayon, poly- ester, PA and acrylic | Overall: 65.8% fibres, 34.2% frag. |  |  | 27 |
| *Thunnus albacares*, South Pacific Ocean | 26 | 5832 | 32 | 1.8 | 14 |  |  | Smallest filter used 63 µm | Overall: 1% < 100 µm, 38% 100-500 µm, 24% 500-1000 µm | Overall: mainly poly- ester, PE and rayon | Overall: 49% frag., 33% fibres, 18% film | Overall: black, blue, white, clear |  | 14 |
| *Thunnus albacares*, South Pacific Ocean | 33 | 4625 | 21.2 | 1.4 | 10 |  |  | Smallest filter used 63 µm | Overall: 1% < 100 µm, 38% 100-500 µm, 24% 500-1000 µm | Overall: mainly poly- ester, PE and rayon | Overall: 49% frag., 33% fibres, 18% film | Overall: black, blue, white, clear |  | 14 |
| *Thunnus albacares*, South Pacific Ocean | 10 | n/a | 70 | 3.1 | 22 |  |  | Smallest filter used 63 µm | Overall: 1% < 100 µm, 38% 100-500 µm, 24% 500-1000 µm | Overall: mainly poly- ester, PE and rayon | Overall: 49% frag., 33% fibres, 18% film | Overall: black, blue, white, clear |  | 14 |
| *Thunnus tonggol*, Malaysia (fish market) | 10 | 195 | 20 |  | n/r |  |  | 149 µm | Overall: minimum 215 µm | Overall: mainly PE | Overall: 67.4% frag., 16.3% fibre, 16.3% film | [continues next page] | Body parts pooled | 7 |
|  |  | Mean | Microplastics concentrations | | | Adj. concentrations | | Size information | | Additional information | | |  |  |
|  | n | weight (g) | % FO | Abundance/ specimen | Totals/ species | Abundance non-fib. | Totals non-fib. | Filter size & LOD | Particle size info | Polymer | Category | Colour | Add. notes | Study ID |
| *Trachurus declivis*, Tasman Sea | 23 | 228 | 0 |  |  | 0 |  | Unknown* |  |  |  |  | Only found microplastics in 1 of 21 species analysed | 25 |
| *Trachurus novaezelandiae*, South Pacific Ocean | 31 | 207 | 3.5 | 1 | 1 |  |  | Smallest filter used 63 µm | Overall: 1% < 100 µm, 38% 100-500 µm, 24% 500-1000 µm | Overall: mainly poly- ester, PE and rayon | Overall: 49% frag., 33% fibres, 18% film | Overall: black, blue, white, clear |  | 14 |
| *Trachurus picturatus*, North East Atlantic | 29 | 238 | 3.4 | 0.03 | 1 | 0.01 | 0.3 | Unknown* | 217-4,810 µm, mean 2,110 µm | Overall: PP, PE, alkyd resin, rayon, poly- ester, PA and acrylic | Overall: 65.8% fibres, 34.2% frag. |  |  | 27 |
| *Trichiurus lepturus*, Portugal (fish market) | 1 | n/r | 0.0 | 0 | 0 | 0 | 0 | Unknown* | 217-4,810 µm, mean 2,110 µm | Overall: PP, PE, alkyd resin, rayon, poly- ester, PA and acrylic | Overall: 65.8% fibres, 34.2% frag. |  |  | 27 |

### Overflow set-up


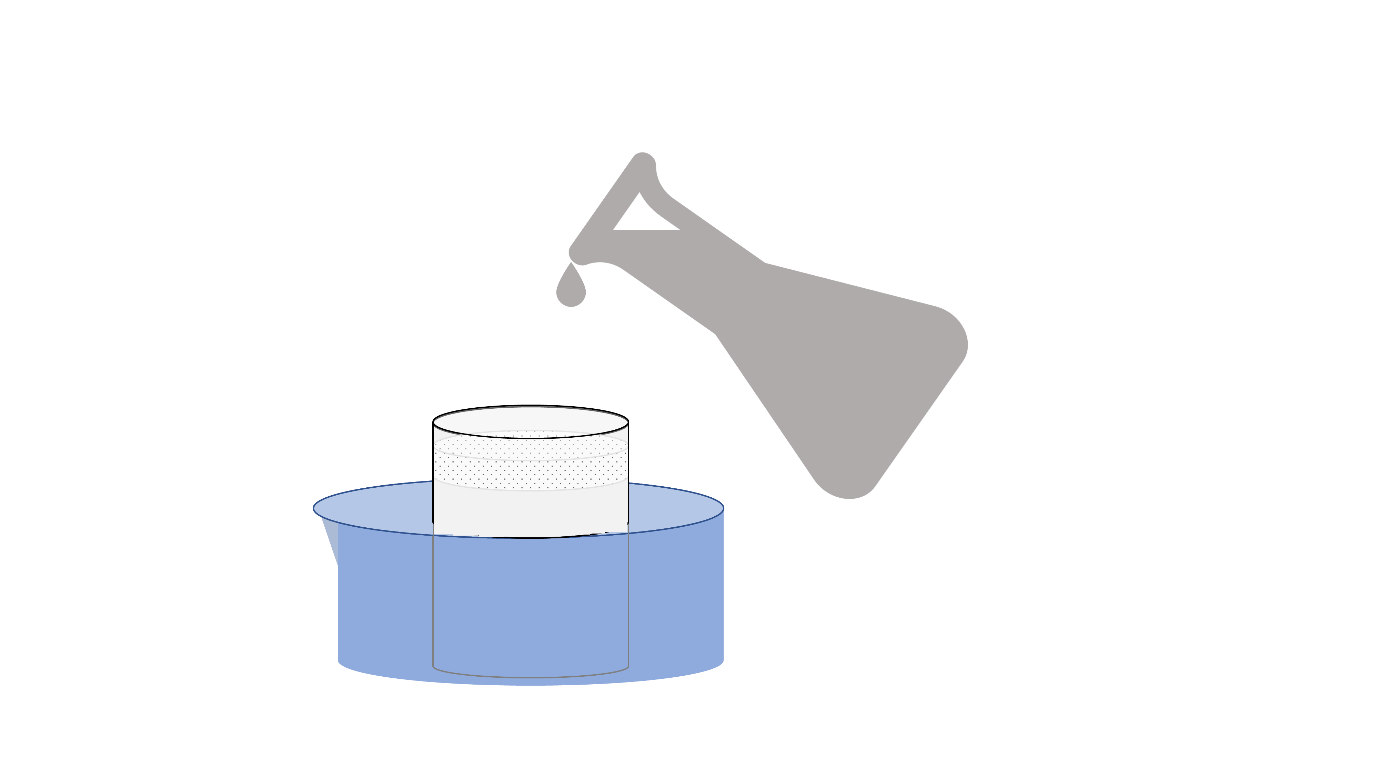


Figure S1 – Overflow NaCl density separation technique: Sampling jars were place inside a beaker to collect density separated microparticles from fishmeal samples (drawing not to scale)

### Supplementary Note - Quantification of theoretical microplastic concentrations in fishmeal

This section details how concentrations of microplastics that theoretically could be expected in fishmeal samples were derived. Only fishmeal-relevant species that are destined whole for production were included (see section ‘whole specimens’ in Table 1 of the main article and Table S2 in this document). Body weights of species were obtained from the reviewed studies or elsewhere (see Table S4 – Body weights of species). To produce 1 kg of fishmeal, 4.5 kg of fish are needed^30,31^.

| Table S4 - Body weights of species | |  |
| --- | --- | --- |
| Species name | weight (g) | Source |
| *Trachurus trachurus* | 153 | Neves *et al.*^27^ |
| *Euthynnus affinis* | 186.6 | Karbalaei *et al.*^7^ |
| *Engraulis encrasicolus* | 12.86 | Compa *et al.*^18^ |
| *Engraulis japonicus* | 4.3 | Sun *et al.*^10^ |
| *Ammodytes personatus* | 7.1 | Sun *et al.*^10^ |
| *Micromesistius poutassou* | 160 | Waterman^32^ |
|  |  |  |
| Simple mean (g) | 87.31 |  |

Based on simple mean weight, number of individual fish needed to produce 1 kg fishmeal: 51.5

Number of microplastics expected in 1 kg of fishmeal based on individual microplastic burden of 0.69 items/individual* 35.6

*chosen the mean microplastic abundance in fishmeal-relevant fish that are used whole only.

Supplementary Table S54 – Recovery rates of dosed microplastics from fishmeal as totals (including standard deviation) and per microplastic type. PS: polystyrene, PP: polypropylene, PET: polyethylene terephthalate, PA: polyamide/nylon. Approximate density of polymers given in brackets^33,34^.

|  | Mean recovery rates (%) | | | | | | |
| --- | --- | --- | --- | --- | --- | --- | --- |
| Fishmeal type | Total | SD | PS (<1.1 g/ml) | PP (<0.9 g/ml) | PET (1.37 g/ml) | PA (1.14 g/ml) | Rayon (1.52 g/ml) |
| Whitefish | 71.3 | 1.2 | 100.0 | 46.7 | 76.7 | 73.3 | 60.0 |
| Sardine/anchovies | 49.3 | 1.2 | 76.7 | 56.7 | 30.0 | 53.3 | 30.0 |

### Supplementary Note - Quantification of fishmeal as microplastics pathway to the sea

|  | non-fibrous only | all microplastics |
| --- | --- | --- |
| Total number of microplastics found in this study in 240 g of fishmeal (after application of the recovery rate of 71.3%) | 17.3 | 29.7 |
| per 1 kg fishmeal | 72.1 | 123.8 |
| per 2.5 million tonnes of fishmeal returned to the sea (amount of fishmeal used per year for marine aquaculture according to Cashion *et al*.^35^) | 180,208,333.3 | 309,375,000.0 |
|  |  |  |
| Mass estimate of microplastic load^ in g based on values obtained by the authors | 8,652.0 | 9,558.8 |
| Mass of microplastic load^ in kg | 8.7 | 9.6 |

| Global mass estimate based on other publications^$^ |  | kg |
| --- | --- | --- |
| Van Sebille *et al.*^36^ (5.4 x 10^-3^ g/microplastic) |  | 1670.6 |
| Galgani in Van Sebille *et al.*^36^ (7.38 x 10^-4^ g/microplastic) |  | 228.3 |

^average fragment weight 4.8 x 10^-5^ g and mean fibre weight 7.0 x 10^-6^ g based on

- fragments: PP (size range 293-3,000 µm), 704 particles weighed 0.0338 g
- fibres: PP (size range 362-7,000 µm, diameter 47 µm), 1287 items weighed 0.0052 g and PA (size range not determined but assumed similar to PP fibres, diameter 100 µm), 30 items weighed 0.0003 g

^$^Weight estimates based on values found in the literature (no distinction made between non-fibrous and fibrous microplastics)

Van Sebille *et al*.^36^ estimate that 15-51 trillion microplastics in the oceans weighing 93,000-236,000 metric tonnes.

| Individual microplastic weight in g | =93000000000/ 15000000000000 | 0.0062 |
| --- | --- | --- |
|  | =236000000000/ 51000000000000 | 0.0046 |
| Mean value in g |  | 0.0054 |

Further value reported by Van Sebille *et al*.^36^ (supplementary data, unpublished data by Galgani):

7.38 x 10^-4^ g/microplastic

Others were excluded due to values based on large microplastics (i.e. pellets) or even particles larger than 5 mm.

Supplementary table S6 - Table S5 - List of species used in fishmeal production based on information in Péron *et al.*^30^, Cashion *et al.*^35^, Shepherd and Jackson^31^ and Newton^37^

| Species | Scientific name |
| --- | --- |
| Sandeels(=Sandlances) | *Ammodytes* spp |
| European eel | *Anguilla anguilla* |
| Meagre | *Argyrosomus regius* |
| Gulf menhaden | *Brevoortia patronus* |
| Atlantic menhaden | *Brevoortia tyrannus* |
| Pacific anchoveta | *Cetengraulis mysticetus* |
| Atlantic herring | *Clupea harengus* |
| Pacific herring | *Clupea pallasii* |
| Black and Caspian Sea sprat | *Clupeonella cultriventris* |
| Pacific saury | *Cololasis saira* |
| Whitefishes nei | *Coregonus* spp |
| Scads nei | *Decapterus* spp |
| European seabass | *Dicentrarchus labrax* |
| Southern African anchovy | *Engraulis capensis* |
| European anchovy | *Engraulis encrasicolus* |
| Japanese anchovy | *Engraulis japonicus* |
| Anchoveta (Peruvian anchovy) | *Engraulis ringens* |
| Kawakawa | *Euthynnus affinis* |
| Pacific cod | *Gadus macrocephalus* |
| Atlantic cod | *Gadus morhua* |
| Alaska pollock (=Walleye poll.) | *Gadus chalcogrammus* |
| Skipjack tuna | *Katsuwonus pelamis* |
| Yellow croaker | *Larimichthys polyactis* |
| Barramundi (=Giant seaperch) | *Lates calcarifer* |
| Emperors (=Scavengers) nei | *Lethrinidae** |
| Patagonian grenadier | *Macruronus magellanicus* |
| Capelin | *Mallotus villosus* |
| Haddock | *Melanogrammus aeglefinus* |
| Hakes | *Merluccius* spp |
| Southern blue whiting | *Micromesistius australis* |
| Blue whiting | *Micromesistius poutassou* |
| Whitemouth croaker | *Micropogonias furnieri* |
| Flathead grey mullet | *Mugil cephalus* |
| Daggertooth pike conger | *Muraenesox cinereus* |
| Threadfin breams nei | *Nemipterus* spp |
| Mote sculpin | *Normanichthys crockeri* |
| Pacific thread herring | *Opisthonema* spp*.* |
| Silver pomfrets nei | *Pampus spp* |
| Okhotsk atka mackerel | *Pleurogrammus azonus* |
| Saithe(=pollock) | *Pollachius virens/Pollachius pollachius* |
| Indian mackerels nei | *Rastrelliger spp* |
| Salmonids | *Salmonidae, incl Oncorhynchus* spp*.^* |
| European pilchard(=sardine) | *Sardina pichardus* |
| Sardinellas nei | *Sardinella* spp |
| California pilchard | *Sardinops caeruleus* |
| Southern African pilchard | *Sardinops ocellatus* |
| Croakers, drums nei | *Sciaenidae#* |
| Blue mackerel | *Scomber australasicus* |
| Chub mackerel | *Scomber japonicus* |
| Atlantic mackerel | *Scomber scombrus* |
| Narrow-barred Spanish mackerel | *Scomberomorus commerson* |
| Seerfishes nei | *Scomberomorus* spp |
| Turbot | *Scophthalmus maximus* |
| Gilthead seabream | *Sparus aurata* |
| European sprat | *Sprattus sprattus* |
| Araucanian herring | *Strangomera bentincki* |
| Albacore | *Thunnus alalunga* |
| Yellowfin tuna | *Thunnus albacares* |
| Bigeye tuna | *Thunnus obesus* |
| Longtail tuna | *Thunnus tonggol* |
| Atlantic bluefin tuna | *Thunnus thynnus* |
| Cape horse mackerel | *Trachurus capensis* |
| Mediterranean horse mackerel | *Trachurus mediterraneus* |
| Jack and horse mackerel nei | *Trachurus* spp |
| Largehead hairtail | *Trichiurus lepturus* |

*according to WoRMS (http://www.marinespecies.org/aphia.php?p=taxdetails&id=151458, accessed 23/03/2020): *Gnathodentex, Gymnocranius, Lethrinus, Monotaxis, Wattsia* and *Pentapodus* species

#according to WoRMS (http://www.marinespecies.org/aphia.php?p=taxdetails&id=125558, accessed 23/03/2020): *Aplodinotus, Argyrosomus, Aspericorvina, Atractoscion, Atrobucca, Austronibea, Bahaba, Bairdiella, Boesemania, Cheilotrema, Chrysochir, Cilus, Collichthys, Corvula, Ctenosciaena, Cynoscion, Daysciaena, Dendrophysa, Elattarchus, Eques, Equetus, Genyonemus, Isopisthus, Johnius, Kathala, Larimichthys, Larimus, Leiostomus, Lonchurus, Macrodon, Macrospinosa, Megalonibea, Menticirrhus, Micropogonias, Miichthys, Miracorvina, Nebris, Nibea, Odontoscion, Otolithes, Otolithoides, Pachypops, Pachyurus, Panna, Paralonchurus, Paranebris, Paranibea, Pareques, Pennahia, Pentheroscion, Petilipinnis, Plagioscion, Pogonias, Protonibea, Protosciaena, Pseudotolithus, Pteroscion, Pterotolithus, Robaloscion, Roncador, Sciaena, Sciaenops, Seriphus, Sonorolux, Stellifer, Totoaba* and *Umbrina* species

^according to WoRMS (http://www.marinespecies.org/aphia.php?p=taxdetails&id=125587, accessed 23/03/2020), direct children only: *Brachymystax, Coregonus, Coregonus, Hucho, Oncorhynchus, Parahucho, Prosopium, Salmo, Salvelinus, Salvethymus, Stenodus* and *Thymallus* species

### References used in Supplementary Information

1. Huang, J. S. *et al.* Microplastic accumulation in fish from Zhanjiang mangrove wetland, South China. *Sci. Total Environ.* **708**, 134839 (2020).

2. Zhang, D. *et al.* Microplastic pollution in water, sediment, and fish from artificial reefs around the Ma’an Archipelago, Shengsi, China. *Sci. Total Environ.* **703**, 134768 (2020).

3. de Vries, A. N., Govoni, D., Árnason, S. H. & Carlsson, P. Microplastic ingestion by fish: Body size, condition factor and gut fullness are not related to the amount of plastics consumed. *Mar. Pollut. Bull.* **151**, 110827 (2020).

4. Lopes, C., Raimundo, J., Caetano, M. & Garrido, S. Microplastic ingestion and diet composition of planktivorous fish. *Limnol. Oceanogr. Lett.* **5**, 103–112 (2020).

5. Kazour, M., Jemaa, S., Issa, C., Khalaf, G. & Amara, R. Microplastics pollution along the Lebanese coast (Eastern Mediterranean Basin): Occurrence in surface water, sediments and biota samples. *Sci. Total Environ.* **696**, 133933 (2019).

6. Hossain, M. S. *et al.* Microplastics in fishes from the Northern Bay of Bengal. *Sci. Total Environ.* **690**, 821–830 (2019).

7. Karbalaei, S. *et al.* Abundance and characteristics of microplastics in commercial marine fish from Malaysia. *Mar. Pollut. Bull.* **148**, 5–15 (2019).

8. Zhang, F. *et al.* Food-web transfer of microplastics between wild caught fish and crustaceans in East China Sea. *Mar. Pollut. Bull.* **146**, 173–182 (2019).

9. Ding, J. *et al.* Microplastics in the coral reef systems from Xisha Islands of South China Sea. *Environ. Sci. Technol.* **53**, 8036–8046 (2019).

10. Sun, X. *et al.* Characteristics and retention of microplastics in the digestive tracts of fish from the Yellow Sea. *Environ. Pollut.* **249**, 878–885 (2019).

11. Giani, D., Baini, M., Galli, M., Casini, S. & Fossi, M. C. Microplastics occurrence in edible fish species (*Mullus barbatus* and *Merluccius merluccius*) collected in three different geographical sub-areas of the Mediterranean Sea. *Mar. Pollut. Bull.* **140**, 129–137 (2019).

12. McGoran, A. R., Cowie, P. R., Clark, P. F., McEvoy, J. P. & Morritt, D. Ingestion of plastic by fish: A comparison of Thames Estuary and Firth of Clyde populations. *Mar. Pollut. Bull.* **137**, 12–23 (2018).

13. Bour, A., Avio, C. G., Gorbi, S., Regoli, F. & Hylland, K. Presence of microplastics in benthic and epibenthic organisms: Influence of habitat, feeding mode and trophic level. *Environ. Pollut.* **243**, 1217–1225 (2018).

14. Markic, A. *et al.* Double trouble in the South Pacific subtropical gyre: Increased plastic ingestion by fish in the oceanic accumulation zone. *Mar. Pollut. Bull.* **136**, 547–564 (2018).

15. Digka, N., Tsangaris, C., Torre, M., Anastasopoulou, A. & Zeri, C. Microplastics in mussels and fish from the Northern Ionian Sea. *Mar. Pollut. Bull.* **135**, 30–40 (2018).

16. Cheung, L. T. O., Lui, C. Y. & Fok, L. Microplastic contamination of wild and captive flathead grey mullet (*Mugil cephalus*). *Int. J. Environ. Res. Public Health* **15**, (2018).

17. Halstead, J. E., Smith, J. A., Carter, E. A., Lay, P. A. & Johnston, E. L. Assessment tools for microplastics and natural fibres ingested by fish in an urbanised estuary. *Environ. Pollut.* **234**, 552–561 (2018).

18. Compa, M., Ventero, A., Iglesias, M. & Deudero, S. Ingestion of microplastics and natural fibres in *Sardina pilchardus* (Walbaum, 1792) and *Engraulis encrasicolus* (Linnaeus, 1758) along the Spanish Mediterranean coast. *Mar. Pollut. Bull.* **128**, 89–96 (2018).

19. Bessa, F. *et al.* Occurrence of microplastics in commercial fish from a natural estuarine environment. *Mar. Pollut. Bull.* **128**, 575–584 (2018).

20. Hermsen, E., Pompe, R., Besseling, E. & Koelmans, A. A. Detection of low numbers of microplastics in North Sea fish using strict quality assurance criteria. *Mar. Pollut. Bull.* **122**, 253–258 (2017).

21. Karlsson, T. M. *et al.* Screening for microplastics in sediment, water, marine invertebrates and fish: Method development and microplastic accumulation. *Mar. Pollut. Bull.* **122**, 403–408 (2017).

22. Murphy, F., Russell, M., Ewins, C. & Quinn, B. The uptake of macroplastic & microplastic by demersal & pelagic fish in the Northeast Atlantic around Scotland. *Mar. Pollut. Bull.* **122**, 353–359 (2017).

23. Ory, N. C., Sobral, P., Ferreira, J. L. & Thiel, M. Amberstripe scad *Decapterus muroadsi* (Carangidae) fish ingest blue microplastics resembling their copepod prey along the coast of Rapa Nui (Easter Island) in the South Pacific subtropical gyre. *Sci. Total Environ.* **586**, 430–437 (2017).

24. Tanaka, K. & Takada, H. Microplastic fragments and microbeads in digestive tracts of planktivorous fish from urban coastal waters. *Sci. Rep.* **6**, 34351 (2016).

25. Cannon, S. M. E., Lavers, J. L. & Figueiredo, B. Plastic ingestion by fish in the Southern Hemisphere: A baseline study and review of methods. *Mar. Pollut. Bull.* **107**, 286–291 (2016).

26. Rummel, C. D. *et al.* Plastic ingestion by pelagic and demersal fish from the North Sea and Baltic Sea. *Mar. Pollut. Bull.* **102**, 134–141 (2016).

27. Neves, D., Sobral, P., Ferreira, J. L. & Pereira, T. T. Ingestion of microplastics by commercial fish off the Portuguese coast. *Mar. Pollut. Bull.* **101**, 119–126 (2015).

28. Collard, F., Gilbert, B., Eppe, G., Parmentier, E. & Das, K. Detection of anthropogenic particles in fish stomachs: An isolation method adapted to identification by Raman spectroscopy. *Arch. Environ. Contam. Toxicol.* **69**, 331–339 (2015).

29. Lusher, A. L., McHugh, M. & Thompson, R. C. Occurrence of microplastics in the gastrointestinal tract of pelagic and demersal fish from the English Channel. *Mar. Pollut. Bull.* **67**, 94–99 (2013).

30. Péron, G., François Mittaine, J. & Le Gallic, B. Where do fishmeal and fish oil products come from? An analysis of the conversion ratios in the global fishmeal industry. *Mar. Policy* **34**, 815–820 (2010).

31. Shepherd, C. J. & Jackson, A. J. Global fishmeal and fish-oil supply: inputs, outputs and markets a. *J. Fish Biol.* **83**, 1046–1066 (2013).

32. Waterman, J. J. *Handling and processing blue whiting*. http://www.fao.org/3/x5952e/x5952e00.htm#Accompanying Notes (2001).

33. Andrady, A. L. Microplastics in the marine environment. *Mar. Pollut. Bull.* **62**, 1596–1605 (2011).

34. Behera, B. K. & Hari, P. K. Using a geometric model to predict woven fabric properties. in *Woven Textile Structure* 30–72 (Elsevier, 2010). doi:10.1533/9781845697815.1.30.

35. Cashion, T., Le Manach, F., Zeller, D. & Pauly, D. Most fish destined for fishmeal production are food-grade fish. *Fish Fish.* **18**, 837–844 (2017).

36. van Sebille, E. *et al.* A global inventory of small floating plastic debris. *Environ. Res. Lett.* **10**, 124006 (2015).

37. Newton, R. W. Assessing environmental sustainability and value addition opportunities for by-products from aquaculture. PhD dissertation. (University of Stirling, 2014).
